# Convergence of human and Old World monkey gut microbiomes demonstrates the importance of human ecology over phylogeny

**DOI:** 10.1186/s13059-019-1807-z

**Published:** 2019-10-08

**Authors:** Katherine R. Amato, Elizabeth K. Mallott, Daniel McDonald, Nathaniel J. Dominy, Tony Goldberg, Joanna E. Lambert, Larissa Swedell, Jessica L. Metcalf, Andres Gomez, Gillian A. O. Britton, Rebecca M. Stumpf, Steven R. Leigh, Rob Knight

**Affiliations:** 1 Department of Anthropology, Northwestern University, 1810 Hinman Ave, Evanston, IL 60208 USA; 2 Department of Pediatrics, University of California San Diego, San Diego, 92093 USA; 3 Center for Microbiome Innovation, University of California San Diego, San Diego, 92093 USA; 4 Department of Anthropology, Dartmouth College, Hanover, 03755 USA; 5 Department of Pathobiological Sciences, University of Wisconsin-Madison, Madison, 53706 USA; 6 Department of Ecology and Evolutionary Biology, University of Colorado Boulder, Boulder, 80302 USA; 7 Department of Anthropology, City University of New York - Queens College, New York, 11367 USA; 8 Department of Animal Sciences, Colorado State University, Fort Collins, 80521 USA; 9 Department of Animal Sciences, University of Minnesota, Minneapolis, 55108 USA; 10 Department of Anthropology, University of Illinois at Urbana-Champaign, Urbana, 61801 USA; 11 Department of Anthropology, University of Colorado Boulder, Boulder, 80302 USA; 12 Department of Computer Science and Engineering, University of California San Diego, San Diego, 92093 USA; 13 Department of Bioengineering, University of California San Diego, San Diego, 92093 USA

**Keywords:** Human gut microbiome, Primate gut microbiome, Cercopithecine, Human evolution

## Abstract

**Background:**

Comparative data from non-human primates provide insight into the processes that shaped the evolution of the human gut microbiome and highlight microbiome traits that differentiate humans from other primates. Here, in an effort to improve our understanding of the human microbiome, we compare gut microbiome composition and functional potential in 14 populations of humans from ten nations and 18 species of wild, non-human primates.

**Results:**

Contrary to expectations from host phylogenetics, we find that human gut microbiome composition and functional potential are more similar to those of cercopithecines, a subfamily of Old World monkey, particularly baboons, than to those of African apes. Additionally, our data reveal more inter-individual variation in gut microbiome functional potential within the human species than across other primate species, suggesting that the human gut microbiome may exhibit more plasticity in response to environmental variation compared to that of other primates.

**Conclusions:**

Given similarities of ancestral human habitats and dietary strategies to those of baboons, these findings suggest that convergent ecologies shaped the gut microbiomes of both humans and cercopithecines, perhaps through environmental exposure to microbes, diet, and/or associated physiological adaptations. Increased inter-individual variation in the human microbiome may be associated with human dietary diversity or the ability of humans to inhabit novel environments. Overall, these findings show that diet, ecology, and physiological adaptations are more important than host-microbe co-diversification in shaping the human microbiome, providing a key foundation for comparative analyses of the role of the microbiome in human biology and health.

**Electronic supplementary material:**

The online version of this article (10.1186/s13059-019-1807-z) contains supplementary material, which is available to authorized users.

## Background

Compared to other primates, humans possess a suite of unique biological and ecological traits [1], including relatively large brains, increased adiposity, and a diet that incorporates domesticated and cooked foods. A wide body of research explores the influences of these traits on each other in the context of human evolutionary trajectories [2–13]. The gut microbiome likely contributes to these dynamics given that it is strongly influenced by host environmental and lifestyle factors and has diverse influences on host physiology and behavior [14–16]. However, clear gaps exist in our knowledge of the processes shaping the human gut microbiome across evolutionary timescales as well as the potential implications for human adaptation.

Comparative analyses of human and non-human primates are powerful tools for exploring the evolutionary history of the human gut microbiome. A better understanding of the primate gut microbiome can provide insight into what aspects of the human microbiome are ancestral and shared among all primates, associated with specific biological or ecological traits throughout the primate phylogeny, or derived and unique to humans. Several studies comparing the gut microbiomes of humans to great apes suggest the importance of host phylogenetic relationships and the co-diversification of microbial lineages with their hosts in shaping the primate microbiome [17–19]. Nevertheless, these analyses are limited to a small number of closely related primate taxa, and a recent systematic examination of data from 18 primate species across the phylogeny reveals that less than 3% of microbial taxa defined by 97% sequence similarity co-diversify with hosts [20]. Although differences in primate microbiome composition and functional potential are strongly associated with host phylogeny, divergences of microbial taxa generally pre-date divergences of the primate species they characterize, suggesting that hosts acquire microbial lineages more ancient than themselves as a result of their ecological niches and associated environmental exposures, physiology, and behavior [20]. In particular, host adaptations of digestive anatomy and physiology to specific dietary niches appear to strongly influence the microbial taxa and gene families a primate possesses [20]. Thus, to the extent that hosts of the same phylogenetic group share physiological dietary adaptations, they will also share gut microbial traits.

These findings are relevant to our perspectives on the human gut microbiome. Although humans are most closely related to great apes, particularly chimpanzees and bonobos (*Pan*), the human ecological niche and associated digestive physiology are distinct from those of great apes. At some point after the divergence of the human lineage from that of *Pan*, our hominin ancestors began to occupy increasingly open and variable habitats, such as wooded grasslands, and to exhibit a broader and more flexible diet [21]. This omnivorous diet included foods high in fat and protein such as meat, but may also have contained underground plant storage organs, particularly those of C4 grasses and sedges [22, 23]. Profound shifts in human diets since the demographic transition toward industrialization mean that few humans consume these ancestral foods in the same quantities today. However, the human diet continues to be extremely diverse both across and within populations [24]. In contrast, great ape species generally inhabit forest ecosystems, and preferentially consume fruit when it is available [25]. Chimpanzees and bonobos, in particular, are described as ripe-fruit specialists, consuming high percentages of fruit even when availability is reduced [26]. These differences in feeding ecology are associated with differences in digestive physiology. For example, salivary amylase expression in chimpanzees is one third of that in humans [27], and both chimpanzees and bonobos have rapid intestinal transit time relative to body mass, which has been associated with their highly frugivorous diet [28, 29].

Instead, humans occupy an ecological niche more similar to that of distantly related cercopithecines (a subfamily of Old World monkey) [30–32]. Cercopithecines inhabit grasslands with varying degrees of woody cover and utilize an omnivorous diet that includes underground plant storage organs of C4 grasses and sedges [33]. It has been previously argued that a subset of cercopithecines, the papionin primates (geladas—*Theropithecus gelada* and baboons—*Papio* spp.), are the best ecological analogues for hominin ancestors [31, 32, 34–45]. For example, in one study, a single female baboon was reported to consume 69 discrete food items from 29 species in one 30-day period [46]. This dietary diversity is reminiscent of humans. Ecological similarities between humans and cercopithecines are also reflected in digestive anatomy and physiology [31, 32]. For instance, humans and baboons have comparable coefficients of gut differentiation—both species exhibit increased small intestinal volume, albeit to different extents [47]. Additionally, like humans, baboons also have high salivary amylase expression [27].

These patterns suggest that common assumptions about the evolution of the human gut microbiome should be tested. Most studies implicitly assume that host-microbe co-diversification processes have dominated the evolution of the human gut microbiome and that the gut microbiomes of other apes are sufficient to provide insight into the evolutionary trajectory of the human gut microbiome [17–19, 48]. However, if, similar to what has been observed in other primates, the evolution of the human gut microbiome has been strongly influenced by host ecological niche and associated digestive physiology, data from other primates, such as cercopithecines, are critical for adequate context. If humans share more gut microbial traits with cercopithecines than with great apes, perspectives on the evolution of the human gut microbiome must shift.

Here, we combine 16S rRNA gene amplicon data and shotgun metagenomic data from 14 populations of industrialized and non-industrialized humans from ten nations [49–53] as well as from 18 species of wild, non-human primates consuming their natural diet [20] (Additional file 1: Table S1) to test the hypothesis that host dietary ecology and digestive physiology influence the human gut microbiome independently of host phylogeny and host-microbe co-diversification processes. Specifically, we assess whether the composition and functional potential of the human gut microbiome are more similar to those of cercopithecines than to those of great apes or vice versa. Additionally, given that humans possess ecological and physiological traits that are unique among primates, we examine whether humans possess microbial traits that are unique compared to both great apes and cercopithecines.

## Results

**Fig. 1:**
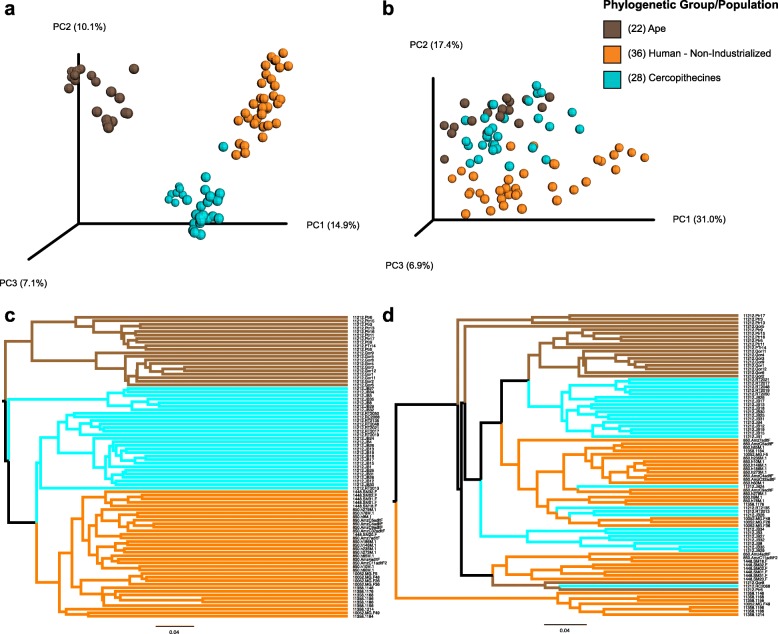
Similarity of gut microbiome composition among humans, apes, and cercopithecines. **a** Principal coordinates analysis (PCoA) plot of 16S rRNA gene amplicon data based on unweighted UniFrac distances. **b** PCoA plot of 16S rRNA gene amplicon data based on weighted UniFrac distances. **c** Consensus unweighted pair group method with arithmetic mean (UPGMA) tree of 16S rRNA gene amplicon data based on unweighted UniFrac distances. **d** Consensus unweighted pair group method with arithmetic mean (UPGMA) tree of 16S rRNA gene amplicon data based on weighted UniFrac distances

We first compared gut microbiome composition and functional potential for both industrialized and non-industrialized humans and all 18 species of wild primates. In agreement with previous reports [51, 52, 54–56], the gut microbiomes of industrialized and non-industrialized human populations differed significantly in both taxonomic composition (16S rRNA gene amplicon data) and functional potential (shotgun metagenomic data; Additional file 2: Figures S1-S7). Gut microbiomes of industrialized populations clustered away from all other primates while gut microbiomes of non-industrialized populations clustered with apes and Old World monkeys (Additional file 2: Figures S1, S2). Given that industrialized humans were clearly outliers and that New World monkeys and lemurs had limited similarities to humans, we removed these samples from all further analyses. Repeating the analysis with only non-industrialized human populations, Old World monkeys, and apes demonstrated that the taxonomic composition of the human gut microbiome (16S rRNA gene amplicon data) was more similar to that of cercopithecines than apes (Fig. 1, Additional file 2: Figure S8). Although the gut microbiome of cercopithecines exhibited higher taxonomic diversity than that of both humans and apes (Additional file 1: Table S2), PERMANOVA confirmed greater differences in gut microbiome taxonomic composition when comparing within-group similarities to between-group similarities for humans and apes (unweighted UniFrac: *F*_1,55_ = 16.0, *r*^2^ = 0.23, *p* < 0.001; weighted UniFrac: *F*_1,55_ = 14.4, *r*^2^ = 0.21, *p* < 0.001) than for humans and cercopithecines (unweighted UniFrac: *F*_1,64_ = 10.5, *r*^2^ = 0.14, *p* < 0.001; weighted UniFrac: *F*_1,64_ = 10.3, *r*^2^ = 0.14, *p* < 0.001). Both the *F* statistic and the *r*^2^ value were larger for the human-ape comparison. When we evaluated how well ecological niche and phylogenetic group were correlated with the ordination of microbiome data, we obtained a similar result. Host ecological niche was more strongly correlated with the microbiome data (unweighted UniFrac: *r*^2^ = 0.49, *p* < 0.001, weighted UniFrac: *r*^2^ = 0.17, *p* < 0.001) than host phylogenetic group was (unweighted UniFrac: *r*^2^ = 0.28, *p* < 0.001, weighted UniFrac: *r*^2^ = 0.04, *p* < 0.001,). Weighted UniFrac distances between humans and cercopithecines were also significantly smaller than distances between humans and apes (*t* = − 9.8, *p* < 0.001). The same patterns emerged when we examined gut microbiome taxonomic composition using shotgun metagenomic data and Bray-Curtis similarity indices (Additional file 2: Figure S9). For example, PERMANOVA comparing within-group to between-group similarity confirmed greater differences in gut microbiome taxonomic composition between humans and apes (Bray-Curtis: *F*_1,29_ = 14.2, *r*^2^ = 0.34, *p* < 0.001) than humans and cercopithecines (Bray-Curtis: *F*_1,40_ = 10.4, *r*^2^ = 0.21, *p* < 0.001).

LEfSe analysis of the 16S rRNA gene amplicon data revealed a similar number of distinguishing taxa between apes and humans and between cercopithecines and humans (Additional file 2: Figure S10). To account for inter-host species variation in relative abundances of specific microbial lineages, we defined the core microbiome as lineages existing in 80% of a group of samples. Using this cutoff, we found more microbial taxa were shared by 80% of humans and cercopithecines than by 80% of humans and apes. Taxa shared between humans and cercopithecines primarily belonged to the Ruminococcaceae and Lachnospiraceae families (Additional file 3: Table S3). Notably, similarities between humans and cercopithecines in this analysis were primarily driven by baboons (Additional file 2: Figures S11, S12; Additional file 3: Table S3; unweighted UniFrac humans vs. baboons: PERMANOVA *F*_1,49_ = 9.6, *r*^2^ = 0.17, *p* < 0.001; weighted UniFrac: *F*_1,49_ = 9.0, *r*^2^ = 0.16, *p* < 0.001).

**Fig. 2:**
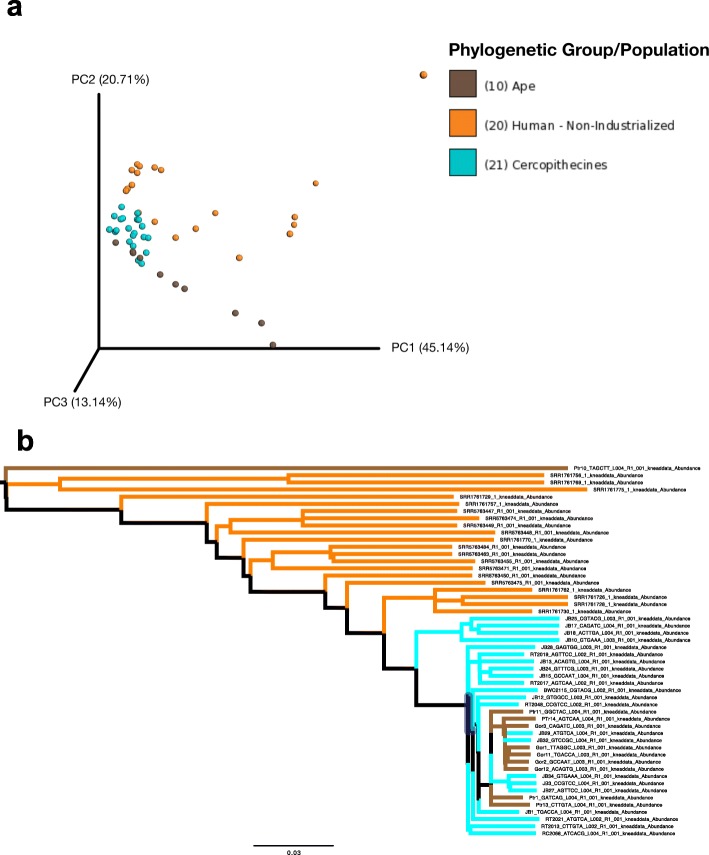
Similarity of gut microbiome functional potential among non-industrialized humans, apes, and cercopithecines. **a** Principal coordinates analysis (PCoA) plot of shotgun metagenomic sequencing data based on Bray-Curtis distances. **b** Consensus unweighted pair group method with arithmetic mean (UPGMA) tree of shotgun metagenomic sequencing data based on unweighted UniFrac distances

When we examined the functional potential of the gut microbiome using shotgun metagenomics to identify relative abundances of MetaCyc Reaction pathways, host ecological niche continued to explain substantial amounts of variation in the data. The overall dataset indicated slightly greater differences between humans and cercopithecines (Fig. 2, Additional file 2: Figure S13; Bray-Curtis: PERMANOVA *F*_1,40_ = 9.7, *r*^2^ = 0.20, *p* < 0.001) than between humans and apes (Bray-Curtis: PERMANOVA *F*_1,29_ = 5.4, *r*^2^ = 0.16, *p* = 0.001) when comparing within-group distances to between-group distances for each pair of host species, as well as somewhat stronger correlations between host phylogeny and gut microbiome functional potential (Bray-Curtis: *r*^2^ = 0.11, *p* = 0.004) than between host ecology and gut microbiome functional potential (Bray-Curtis ecological group: *r*^2^ = 0.07, *p* = 0.05). However, between-group Bray-Curtis distances for humans and cercopithecines were significantly smaller than distances between humans and apes (*t* = − 4.1, *p* = 0.002).

Additionally, LEfSe analysis indicated that humans and apes were differentiated by more functional pathways than humans and cercopithecines (Additional file 2: Figure S14). Core microbiome analysis using the same cutoff as described for the 16S rRNA gene amplicon data indicated that 96 MetaCyc Reaction pathways were shared between 80% of humans and apes sampled while 122 were shared between humans and cercopithecines (Additional file 4: Table S4). All pathways shared by humans and apes were also shared by cercopithecines, while 26 pathways were uniquely shared by humans and cercopithecines and not found in apes. These pathways were predominately associated with unclassified microbial taxa and *Faecalibacterium prausnitzii* and are involved in processes such as amino acid biosynthesis and starch and sugar degradation. As observed with the 16S rRNA gene amplicon data, shared patterns in potential function between humans and cercopithecines can largely be attributed to baboons (Additional file 2: Figure S15, S16; Additional file 4: Table S4; Bray-Curtis humans vs. baboons: PERMANOVA *F*_1,29_ = 4.2, *r*^2^ = 0.13, *p* < 0.00).

Using the shotgun metagenomic data to examine the relative abundances of carbohydrate-active enzymes (CAZymes) provided additional functional insight. The overall CAZyme dataset indicated similar differences between humans and cercopithecines (Bray-Curtis: *F*_1,39_ = 11.9, *r*^2^ = 0.24, *p* < 0.001) and humans and apes (Bray-Curtis: *F*_1,28_ = 7.8, *r*^2^ = 0.22, *p* = 0.004), as well as similar correlations between CAZyme data and host phylogeny (Bray-Curtis: *r*^2^ = 0.10, *p* = 0.0) and ecological niche (Bray-Curtis: *r*^2^ = 0.06, *p* = 0.03). Bray-Curtis distances between humans and cercopithecines were also similar between humans and apes (*t* = − 1.8, *p* > 0.05). LEfSe analysis indicated a similar number of CAZymes distinguishing humans and apes and humans and cercopithecines. These patterns were a result of enzymes for plant structural carbohydrate breakdown, which were enriched in both apes and cercopithecines compared to humans (Additional file 2: Figure S17). However, there were fewer differences between humans and baboons than between humans and both apes and cercopithecines more broadly (Bray-Curtis: *F*_1,28_ = 5.6, *r*^2^ = 0.17, *p* = 0.01, Additional file 2: Figure S18). Results were the same when only carbohydrate binding molecules or glycoside hydrolases are considered, suggesting that convergence of microbial CAZymes for humans and baboons is not limited to a specific subset of CAZymes.

Finally, our data also revealed unique human microbiome traits compared to both cercopithecines and apes. Humans were enriched for 11 microbial taxa, including *Helicobacter pylori* and *Bacteroides fragilis*, and depleted in 20 compared to both cercopithecines and apes (Additional file 2: Figure S19). Humans were also enriched for 44 MetaCyc pathways, including many unclassified pathways associated with *Butyrivibrio crossotus* and *Streptococcus salivarius*, and depleted in 30, many of which were associated with nutrient synthesis (Additional file 2: Figure S20). These differences were stronger when we examined CAZymes. Humans were enriched for 11 CAZymes and depleted in 102 (Additional file 2: Figure S21). Additionally, tests of beta dispersion indicated that humans had similar inter-individual variation in the taxonomic composition of their gut microbiomes as both cercopithecines and apes (Additional file 2: Figure S22, ANOVA *F*_1,83_ = 1.4, *p* = 0.2), but more inter-individual variation in functional potential (Additional file 2: Figure S22; ANOVA *F*_1,49_ = 15.2, *p* < 0.001). These results suggest that there is more functional variation represented within the human microbiome than within or across closely related primate species.

## Discussion

Our data demonstrate that the human gut microbiome diverges from closely genetically related apes and converges with cercopithecines both taxonomically and functionally. This finding provides insight into the mechanisms by which the human gut microbiome emerged. Given that the human dietary niche and associated physiological adaptations are more similar to those of cercopithecines (especially baboons) than apes [27–32, 34–45], our results highlight the importance of human ecology and digestive physiology in shaping the gut microbiome. As observed in other contexts [20], co-diversification of host and microbes does not appear to be a dominant process shaping the assembly of the human gut microbiome. Instead, both humans and other primates appear to acquire microbial lineages more ancient than themselves through selection by a suite of host ecological and physiological traits. This process may in part explain previously reported greater-than-expected differences in the human and ape gut microbiomes based on host phylogenetic distances [18]. Assuming acquired microbial lineages are maintained in host populations across generations as a result of some physiological benefit that ultimately affects host fitness [57, 58], exploring the taxonomy and function of these lineages, as well as their impact on human physiology, will provide critical insight into human biology and health.

It is also important to note that, in addition to host ecology and physiology, host biogeography may contribute to the observed convergence of human and non-human primate microbiomes. Humans are the most widespread primates on the planet, and cercopithecines, including baboons and macaques (*Macaca* spp.), also have large geographic distributions, which overlap substantially with those of humans. This physical proximity increases the potential for microbial exchange—and therefore microbial similarity—between humans, baboons, and macaques [59–61]. A current paucity of wild primate microbiome data makes it difficult to test the extent to which biogeography influences the human microbiome. However, if increased geographical overlap leads to gut microbiome convergence, we would expect cercopithecine species that are commonly sympatric with humans to have the most similar gut microbiomes to those of humans. For example, a subset of macaque and baboon species are sometimes considered “weed” species given their ability to thrive in anthropogenically impacted habitats [42, 62]. If biogeography is a key factor shaping the primate microbiome, “weed” species should share more microbiome traits with humans compared to other macaque and baboon species that co-exist with humans less commonly.

Additionally, despite detecting the strongest microbiome similarities between humans and cercopithecines, we did identify a substantial number of shared microbiome traits between humans and apes. Whether these shared microbiome traits are a result of co-diversification of a subset of microbial lineages with their hosts, or a subset of shared host physiological traits that select for similar microbial lineages remains to be seen. As described above, further exploration of their taxonomy and function, as well as their influence on human physiology, is necessary to understand their relevance to human biology and health.

Interestingly, the similarities we detected between humans and both apes and cercopithecines indicate that the human microbiome may represent a “hybrid” of primate microbiomes. How this hybrid microbiome emerged remains unclear. However, we found that gut microbiome metabolic functional potential was more strongly influenced by human ecology, while human phylogeny had a stronger effect on overall microbiome gene content. Therefore, it is possible that microbial lineages with genes to interface directly with the host immune system, for example, co-diversified with humans while microbial lineages that perform host metabolic services were acquired more recently through exposure. Alternatively, given that gut microbiome taxonomic composition is most similar between humans and cercopithecines, it could be that humans acquired microbial lineages that provided specific metabolic services to hosts, which subsequently evolved or acquired genes necessary to interact with the host immune system. Additional research, including controlled manipulations and improved gene annotations, is necessary to isolate these processes. Regardless of how it emerged though, exploring the potential contributions of this microbial phenotype to human evolution—perhaps by conferring functions that allowed humans to live as apes in a cercopithecine habitat—has the potential to transform our perspectives on human biology and evolution.

Likewise, our data clearly indicate that the human gut microbiome exhibits unique traits that are not present in other primates. Compared to the gut microbiomes of apes and cercopithecines, the human gut microbiome was enriched for a subset microbial taxa and functions, including some that have been clearly linked to human health [63–66]. Inter-individual variation in gut microbiome functional potential was also greater in humans than within and across closely related primate species. Patterns of inter-individual variation in the human gut microbiome have been explored elsewhere in the literature, and increased variation in industrialized populations has been associated with low-fiber diets, frequent antibiotic use, and even differential exposures during early life [51, 67–69]. While differences between non-industrialized human populations and non-human primates could be a result of some of these factors, they also suggest that some degree of microbiome flexibility is innate to all humans. We speculate that this flexibility could have facilitated the diversification of the human dietary niche across evolutionary time, which would have supported human population expansion into new habitats and, ultimately, human evolutionary success [58, 70].

What incited this microbiome flexibility remains unknown. However, human ecology may again provide clues. Cooking is one of the hallmarks of human diet and evolution [10] and represents one of the first food-processing techniques that facilitated hominin utilization of a wider diversity of food items, including plant underground storage organs, by increasing digestibility [11]. Additionally, human utilization of fermented foods appears to be a more ancient practice that also would have improved food digestibility [71, 72]. Interestingly, cercopithecines possess unique cheek pouches that are utilized, in part, to predigest food [73, 74]. Therefore, food fermentation and cooking, among other factors, could have triggered shifts in the hominin gut microbiome that made it simultaneously more similar to those of cercopithecines and unique among primates, as well as extremely flexible. If this were the case, the combined nutritional benefits afforded to human ancestors occupying variable environments and/or migrating to new environments are likely to have been great.

We acknowledge that this study has limitations. First, we have combined existing datasets generated by multiple laboratories, which could introduce technical bias into the results. The majority of data presented here [20, 53] were generated by a single lab using the same protocols, and we screened studies that were included to ensure similar methods were utilized to generate the data. We detected no evidence of the effects of sample preservative, sequencing run, and other technical variables on our final data. Additionally, shotgun metagenomic data, which are less susceptible to some of the technical confounds that influence 16S rRNA gene amplicon data, confirmed patterns observed in the 16S rRNA gene amplicon data.

Second, humans are represented by many more populations than any other species of primate included in the analysis. While we agree that more extensive sampling of wild primates is warranted, it is unlikely to strongly impact the findings presented here. Previous research has shown that, barring captivity, intra-host species microbiome variation across time and space is much smaller than inter-host species microbiome variation for wild primates [20]. As a result, while specific microbial taxa and genes that distinguish these host groups may shift, our overall findings are unlikely to change. Therefore, the addition of more non-human primate data may affect the specific microbial taxa and genes shared among hosts, but it is unlikely to alter broad patterns of microbiome similarity among hosts. In fact, another recent dataset using distinct samples and methods detected a similar pattern to the one we report here [75], suggesting our findings are robust and repeatable. With regard to patterns of inter-individual variation, it is also important to note that even when we include multiple species and genera in the non-human primate group, the amount of inter-host species microbiome variation observed is less than the amount observed within the human species. These patterns suggest that the human gut microbiome may be uniquely plastic in response to host local environment (and associated factors such as diet). Subsequent studies that include more geographically diverse non-human primate populations and/or integrate experimental manipulations of diet should further investigate this hypothesis.

## Conclusions

In conclusion, the human gut microbiome departs from phylogenetic patterns within the order Primates, diverging from apes and exhibiting the greatest similarities with cercopithecines such as baboons. These findings emphasize that human diet, ecology, and physiological adaptations are more important for shaping the gut microbiome than host-microbe co-diversification. Nevertheless, humans possess a range of unique physiological and behavioral characteristics, and the gut microbiome appears to be no exception. It is uniquely enriched for specific microbial taxa and functional pathways and exhibits increased inter-individual variation. While the physiological consequences of this finding for hosts have yet to be fully explored, it re-situates the human gut microbiome within a broader evolutionary framework, offering new insight into the role of the gut microbiome in human biology and health. In this context, continued comparative microbiome research with non-human primates will be critical to the field of medicine as well as human evolutionary biology.

## Methods

### 16S rRNA gene amplicon data generation

Data from the American Gut manuscript [53] package were obtained on September 11, 2017 from ftp://ftp.microbio.me. Studies obtained from Qiita were the folivorous primate gut (Qiita ID: 11212), Yanomami (Qiita ID: 10052), Peruvian gut (Qiita ID: 1448), Global gut (Qiita ID: 850), and the Hadza (Qiita ID: 11358). Sample origins are described in Table S1 (Additional file 1), and more detail is available in the original publications. All sequence data were run through Deblur v1.0.2 [76] using a trim length of 100 nt (the read length of study 850), with the minimum number of reads set to 0 to avoid introducing a per-study effect for low abundant sOTUs. Blooms as determined from Amir et al. [77] were removed from the data using QIIME [78], as were singletons and doubletons. Samples with fewer than 1000 reads were removed. The remaining Deblur sOTUs were inserted into Greengenes 13_8 [79] using SEPP [80]. Taxonomy was assigned using the Naive Bayes classifier in QIIME 2017.4 against Greengenes 13_8. Ten adults (humans: 18–36 years; primates: species-specific) were pseudo-randomly chosen by hand from each host species/human population (unless fewer samples were available) with samples included from a range of ethnic backgrounds when relevant. Sequences corresponding to chloroplasts and mitochondria were removed. The data were then rarefied to 9870 sequences per sample. Chao1, observed species, and Faith’s phylogenetic distance diversity indices were calculated for each sample using alpha_diversity.py. Weighted and unweighted UniFrac distances were calculated among samples using beta_diversity_through_plots, and all data were visualized using a principal coordinate (PCoA) plot generated by Emperor as well as in a consensus UPGMA tree (out of 1000 permutations) built using jackknifed_beta_diversity.py with data rarefied to 9870 sequences per sample.

### Shotgun metagenomic data generation

Shotgun data from the American Gut project (Qiita ID: 10317) and Folivorous primate gut (Qiita ID: 11212) were obtained from Qiita. Shotgun data for the Hadza (PRJNA392180) and the Peruvian gut (PRJNA268964) were obtained from NCBI (www.ncbi.nlm.nih.gov/sra). Again, basic sample information is listed in Additional file 1: Table S1 with additional details in the original publications. Raw metagenomic sequences were trimmed using a 4-bp sliding window with an average quality score of 20 in Trimmomatic [81], and reads that mapped to the human genome (hg19) were removed in KneadData (v0.6.1). Individual samples were analyzed in HUMAnN2 (v0.11.1) [82], using the default options, with the exception of using the UniRef50 protein database as the translated search database. Pathway abundance tables were joined, normalized using relative abundance, and then split into unstratified and stratified tables in HUMAnN2. Gene family tables were joined, normalized by copies per million, regrouped into KEGG Orthogroups, and then split into unstratified and stratified tables in HUMAnN2. We also used MetaPhlAn to extract species-level OTU tables from our shotgun data. Additionally, to describe the relative abundance of carbohydrate-active enzymes associated with each sample, quality-filtered human, ape, and cercopithecine sequences were translated using EMBOSS transeq and were aligned against the dbCAN database (http://csbl.bmb.uga.edu/dbCAN/) using the hmmscan tool in HMMER v.3.2.1 (hmmer.org). The domain table output was filtered to remove hits with an e-value greater than 1.0e−3 and coverage less than 30% using a custom script (https://github.com/emallott/hmmscan_parser). After converting stratified pathway abundance tables and CAZyme abundance tables to biom format, QIIME (v1.9.1) [78] was used for beta diversity analysis and PCoA plots were created using Bray-Curtis distances.

### Statistical analyses

Given the clustering patterns observed in the ordination plots, all but those samples corresponding to apes, cercopithecines, and humans were filtered out for formal analysis. Differences between the microbiome taxonomic composition and functional potential of industrialized and non-industrialized humans were evaluated using the *adonis* function from the *vegan* package (v2.4-6) in R (v3.4.3), with 5000 permutations with alpha = 0.05. Differences in taxonomic diversity were evaluated using an ANOVA with alpha = 0.05. An LDA-type analysis was run and visualized in LEfSe [83], to determine which microbial taxa and genes distinguished non-industrialized and industrialized humans. We evaluated the average distance to the group centroid for humans compared to cercopithecines and primates using the *betadisper* function in the *vegan* package. Given strong differences between the two human groups, industrialized humans were excluded for the majority of the remaining analyses, except where indicated.

The strength of host phylogeny as a predictor of gut microbiome composition was tested for humans vs. apes and humans vs. cercopithecines using the *adonis* function from the *vegan* package (v2.4-6) in R (v3.4.3), with 5000 permutations, both with and without industrialized human populations. We compared models using the reported *F* statistic, which reflects the variance between treatments divided by the variance within treatments, and the *r*^2^ value, which indicates the percentage of variation in the distance matrix explained by the variable of interest by dividing the sums of squares of each variable by the total sums of squares. We also directly compared the goodness of fit of vectors explaining host phylogeny (ape vs. monkey) and host ecological group (forest vs. savannah) on the ordination using the *envfit* function from the *vegan* package. We tested for differences in average inter-group distances between humans and apes and humans and cercopithecines using two-sided Student’s two-sample *t* test. We also performed the same tests on the taxonomic data generated from the shotgun sequences by MetaPhlAn. An LDA-type analysis was run for the 16S data and visualized in LEfSe [83], to determine which microbial taxa distinguish humans compared with apes and humans compared with cercopithecines. Features with a logarithmic LDA score of > 3.0 using default parameters were kept. Microbes shared by 80% of all human and ape samples and all human and cercopithecine samples were determined using compute_core.py. We relaxed the core microbiome definition of 100% prevalence and chose an 80% cutoff since we expected relative abundances of sOTUs to be distinct across host species, and this cutoff has been previously shown to detect core microbial taxa with potentially low abundances across distinct microbial communities [84]. Indeed, no shared microbial taxa were detected in more than 90% of human and ape samples. Finally, we evaluated the average distance to the group centroid for humans compared to cercopithecines and primates using the *betadisper* function in the *vegan* package. We tested for differences in these distances using an ANOVA. In all cases, QIIME v1.9.1 was used unless otherwise noted.

As described for the 16S data, analyses were run on pathway abundance and gene family tables, as well as CAZyme abundance tables, containing cercopithecine, ape, and non-industrialized human samples only. Data were visualized in a PCoA plot using Emperor as well as in a consensus UPGMA tree (out of 1000 permutations) built using jackknifed_beta_diversity.py with data rarefied to 22,000 sequences per sample. Average pathway richness and CAZyme richness was calculated for all phylogenetic groups. Using the *adonis* function from the *vegan* package (v2.4-6) in R (v3.4.3), PERMANOVAs were run to assess the effect of phylogenetic group on differences in pathway abundance and gene families based on Bray-Curtis distance matrices, as well as CAZyme abundance based on Bray-Curtis distance matrices, comparing humans to apes and humans to cercopithecines. The amount of microbial variation correlated with host phylogenetic group (ape vs. monkey) was compared to the amount of microbial variation correlation with the host ecological group (forest vs. savannah) using the *envfit* function from the *vegan* package. We also executed two-tailed Student’s two-sample *t* tests to compare the average inter-group distances between humans and cercopithecines and humans and apes for both pathway and CAZyme data. An LDA-type analysis was run and visualized in LEfSe, to determine which pathways and CAZymes distinguish humans compared with apes and humans compared with cercopithecines. Features with a logarithmic LDA score of > 3.0 using default parameters were kept. Shared pathways and CAZymes between 80% of humans and apes and between 80% of humans and cercopithecines were determined using compute_core.py. We used the same cutoff as the 16S rRNA data for consistency. We also evaluated the average distance to the group centroid for humans compared to cercopithecines and primates using the *betadisper* function in the *vegan* package.

## Additional files

Additional file 1: **Table S1.** Data sources for samples Table S2. Gut microbial diversity of humans, apes, and cercopithecines. (DOCX 123 kb)

Additional file 2: **Figure S1.** Similarity of gut microbiome composition among non-human primates and humans. Figure S2. Similarity of gut microbiome functional potential among non-human primates and humans. Figure S3. Dissimilarity of gut microbiome composition among industrialized and non-industrialized humans. Figure S4. Dissimilarity of gut microbiome functional potential among industrialized and non-industrialized humans. Figure S5. Differences in the taxonomic diversity of industrialized and non-industrialized human gut microbiomes. Figure S6. Differences in the gut microbiome of industrialized and non-industrialized humans. Figure S7. Interindividual variation in the gut microbiome of industrialized and non-industrialized humans. Figure S8. Similarity of gut microbiome composition among industrialized and non-industrialized humans, apes, and cercopithecines. Figure S9. Similarity of gut microbiome composition among non-industrialized humans, apes, and cercopithecines. Figure S10. Microbial taxa distinguishing non-industrialized humans and apes, non-industrialized humans and cercopithecines. Figure S11. Role of baboons in driving similarity of gut microbiome composition among non-industrialized humans, apes, and cercopithecines. Figure S12. Microbial taxa distinguishing non-industrialized humans and baboons. Figure S13. Similarity of gut microbiome functional potential among all humans, apes, and cercopithecines. Figure S14. Metacyc pathways distinguishing non-industrialized humans and apes, non-industrialized humans and cercopithecines. Figure S15. Role of baboons in driving similarity of gut microbiome functional potential among non-industrialized humans, apes, and cercopithecines. Figure S17. Carbohydrate-active enzymes distinguishing non-industrialized humans and apes, non-industrialized humans and cercopithecines. Figure S18. Carbohydrate-active enzymes distinguishing non-industrialized humans and baboons. Figure S19. Microbial taxa distinguishing non-industrialized humans from both apes and cercopithecines. Figure S20. Metacyc pathways distinguishing non-industrialized humans from both apes and cercopithecines. Figure S21. Carbohydrate-active enzymes distinguishing non-industrialized humans from both apes and cercopithecines. Figure S22. Interindividual variation in the gut microbiome of non-industrialized humans and apes/cerocpithecines. (DOCX 23416 kb)

Additional file 3: **Table S3.** Shared gut microbial taxa between host taxonomic groups. (XLSX 56 kb)

Additional file 4: **Table S4.** Shared Metacyc pathways between host taxonomic groups. (XLSX 16 kb)

Additional file 5: Mapping file for 16S data. (TXT 31 kb)

Additional file 6: Mapping file for shotgun data. (TXT 26 kb)

Additional file 7: Review history. (DOCX 33 kb)

## Data Availability

All data are from previously published papers [20, 49–53] and are available from the EBI with the following Project IDs: ERP104379 [85], ERP008799 [86], ERP008694 [87], ERP014589 [88], PRJEB3079 [89], ERP109605 [90], ERP012803 [91]. They are also publicly available on Qiita [92] with the following Qiita IDs: 11212, 10052, 1448, 850, 11358, 10317. All analysis code is available on Github [93] and can also be accessed at zenodo [94]. Relevant mapping files are included in Additional files 5 and 6.
