# Supplementary material for: Convergence of human and Old World monkey gut microbiomes demonstrates the importance of human ecology over phylogeny
Source: Genome Biol. 2019 Oct 8;20:201. doi: 10.1186/s13059-019-1807-z (PMC6781418; doi:10.1186/s13059-019-1807-z)
Supplement: Supplementary file 1 — Table S1. Data sources for samples Table S2. Gut microbial diversity of humans, apes, and cercopithecines. (DOCX 123 kb) [file 13059_2019_1807_MOESM1_ESM.docx]

**Table S1: Data sources for samples from distinct primate species and human populations.**

| **Species** | **Phylogeneic Group** | **Continent** | | **Country** | | **Lifestyle** | | **Source** | |
| --- | --- | --- | --- | --- | --- | --- | --- | --- | --- |
| *Alouatta caraya* | New World Monkey | South America | Argentina | | na | | Amato et al. 2019 | |  |
| *Alouatta palliata* | New World Monkey | North America | Nicaragua | | na | | Amato et al. 2019 | |  |
| *Alouatta pigra* | New World Monkey | North America | Mexico | | na | | Amato et al. 2019 | |  |
| *Alouatta seniculus* | New World Monkey | South America | Colombia | | na | | Amato et al. 2019 | |  |
| *Ateles belzebuth* | New World Monkey | South America | Ecuador | | na | | Amato et al. 2019 | |  |
| *Ateles hybridus* | New World Monkey | South America | Colombia | | na | | Amato et al. 2019 | |  |
| *Cercopithecus ascanius* | Old World Monkey - Cercopithecine | Africa | Uganda | | na | | Amato et al. 2019 | |  |
| *Colobus guereza* | Old World Monkey | Africa | Uganda | | na | | Amato et al. 2019 | |  |
| *Eulemur rubriventer* | Lemur | Africa | Madagascar | | na | | Amato et al. 2019 | |  |
| *Gorilla gorilla* | Ape | Africa | CAR | | na | | Amato et al. 2019 | |  |
| *Homo sapiens* | Human | Africa | Tanzania | | hunter-gatherer | | Smits et al. 2017 | |  |
| *Homo sapiens* | Human | Australia | Australia | | urban | | McDonald et al. 2018 | |  |
| *Homo sapiens* | Human | Africa | Malawi | | remote farmer | | Yatsunenko et al. 2012 | |  |
| *Homo sapiens* | Human | South America | Peru | | hunter-gatherer | | Obregon-Tito et al. 2015 | |  |
| *Homo sapiens* | Human | South America | Peru | | remote farmer | | Obregon-Tito et al. 2015 | |  |
| *Homo sapiens* | Human | Europe | Sweden | | urban | | McDonald et al. 2018 | |  |
| *Homo sapiens* | Human | Europe | Switzerland | | urban | | McDonald et al. 2018 | |  |
| *Homo sapiens* | Human | Asia | Thailand | | urban | | McDonald et al. 2018 | |  |
| *Homo sapiens* | Human | Europe | United Kingdom | | urban | | McDonald et al. 2018 | |  |
| *Homo sapiens* | Human | North America | USA | | urban | | Yatsunenko et al. 2012 | |  |
| *Homo sapiens* | Human | North America | USA | | urban | | Obregon-Tito et al. 2015 | |  |
| *Homo sapiens* | Human | North America | USA | | urban | | McDonald et al. 2018 | |  |
| *Homo sapiens* | Human | South America | Venezuela | | remote farmer | | Yatsunenko et al. 2012 | |  |
| *Homo sapiens* | Human | South America | Venezuela | | hunter-gatherer | | Clemente et al. 2015 | |  |
| *Lagothrix lagotricha* | New World Monkey | South America | Ecuador | | na | | Amato et al. 2019 | |  |
| *Lemur catta* | Lemur | Africa | Madagascar | | na | | Amato et al. 2019 | |  |
| *Pan troglodytes* | Ape | Africa | Uganda | | na | | Amato et al. 2019 | |  |
| *Papio anubis* | Old World Monkey - Cercopithecine | Africa | Ethiopia | | na | | Amato et al. 2019 | |  |
| *Papio hamadryas* | Old World Monkey - Cercopithecine | Africa | Ethiopia | | na | | Amato et al. 2019 | |  |
| *Piliocolobus badius* | Old World Monkey | Africa | Uganda | | na | | Amato et al. 2019 | |  |
| *Propithecus verreauxi* | Lemur | Africa | Madagascar | | na | | Amato et al. 2019 | |  |
| *Theropithecus gelada* | Old World Monkey - Cercopithecine | Africa | Ethiopia | | na | | Amato et al. 2019 | |  |

**Table S2: Gut microbial diversity of humans, apes, and cercopithecines.**

|  | **Observed OTUs** | | | **Chao1** | |  | | **PD Whole Tree** | | |
| --- | --- | --- | --- | --- | --- | --- | --- | --- | --- | --- |
|  | AVG | SD | AVG | | SD | | AVG | | SD |  |
| Industrialized Humans | 174.4 | 57.2 | 195.2 | | 62.6 | | 18.8 | | 5.0 |  |
| Non-industrialized Humans | 318.8 | 79.2 | 380.9 | | 98.7 | | 31.1 | | 6.8 |  |
| Apes | 394.3 | 60.8 | 428.1 | | 65.3 | | 46.9 | | 6.4 |  |
| Cercopithecines | 492.8 | 68.3 | 540.7 | | 75.3 | | 47.8 | | 5.5 |  |
