# Supplementary material for: Convergence of human and Old World monkey gut microbiomes demonstrates the importance of human ecology over phylogeny
Source: Genome Biol. 2019 Oct 8;20:201. doi: 10.1186/s13059-019-1807-z (PMC6781418; doi:10.1186/s13059-019-1807-z)
Supplement: Supplementary file 2 — Figure S1. Similarity of gut microbiome composition among non-human primates and humans. Figure S2. Similarity of gut microbiome functional potential among non-human primates and humans. Figure S3. Dissimilarity of gut microbiome composition among industrialized and non-industrialized humans. Figure S4. Dissimilarity of gut microbiome functional potential among industrialized and non-industrialized humans. Figure S5. Differences in the taxonomic diversity of industrialized and non-industrialized human gut microbiomes. Figure S6. Differences in the gut microbiome of industrialized and non-industrialized humans. Figure S7. Interindividual variation in the gut microbiome of industrialized and non-industrialized humans. Figure S8. Similarity of gut microbiome composition among industrialized and non-industrialized humans, apes, and cercopithecines. Figure S9. Similarity of gut microbiome composition among non-industrialized humans, apes, and cercopithecines. Figure S10. Microbial taxa distinguishing non-industrialized humans and apes, non-industrialized humans and cercopithecines. Figure S11. Role of baboons in driving similarity of gut microbiome composition among non-industrialized humans, apes, and cercopithecines. Figure S12. Microbial taxa distinguishing non-industrialized humans and baboons. Figure S13. Similarity of gut microbiome functional potential among all humans, apes, and cercopithecines. Figure S14. Metacyc pathways distinguishing non-industrialized humans and apes, non-industrialized humans and cercopithecines. Figure S15. Role of baboons in driving similarity of gut microbiome functional potential among non-industrialized humans, apes, and cercopithecines. Figure S17. Carbohydrate-active enzymes distinguishing non-industrialized humans and apes, non-industrialized humans and cercopithecines. Figure S18. Carbohydrate-active enzymes distinguishing non-industrialized humans and baboons. Figure S19. Microbial taxa distinguishing non-industrialized humans fr [file 13059_2019_1807_MOESM2_ESM.docx]

**Fig. S1. Similarity of gut microbiome composition among non-human primates and humans.** **a** Principal coordinates analysis (PCoA) plot of 16S rRNA gene sequencing data based on unweighted UniFrac distances for 18 species of wild non-human primates and 14 populations of humans. **b** PCoA plot of 16S rRNA gene sequencing data based on weighted UniFrac distances. New World monkeys are labeled in shades of green, lemurs in shades of purple, Old World monkeys in shades of blue, apes in shades of brown, non-industrialized human populations in shades of orange, industrialized populations of humans in shades of red.

**Fig. S2.** **Similarity of gut microbiome functional potential among non-human primates and humans.** Principal coordinates analysis (PCoA) plot of shotgun metagenomic sequencing data based on Bray-Curtis distances for 18 species of wild non-human primates and 14 populations of humans. New World monkeys are labeled in shades of green, lemurs in shades of purple, Old World monkeys in shades of blue, apes in shades of brown, non-industrialized human populations in shades of orange, industrialized populations of humans in shades of red.

**Fig. S3. Dissimilarity of gut microbiome composition among industrialized and non-industrialized humans.** **a** Principal coordinates analysis (PCoA) plot of 16S rRNA gene sequencing data based on unweighted UniFrac distances for 14 populations of humans (PERMANOVA F_1,88_ = 20.6, r^2^ = 0.19, p < 0.001). **b** PCoA plot of 16S rRNA gene sequencing data based on weighted UniFrac distances (PERMANOVA F_1,88_ = 14.5, r^2^ = 0.14, p < 0.001)

**Fig. S4. Dissimilarity of gut microbiome function among industrialized and non-industrialized humans.** Principal coordinates analysis (PCoA) plot of shotgun metagenomic sequencing data based on Bray-Curtis distances for 14 populations of humans (PERMANOVA F_1,44_ = 13.7, r^2^ = 0.24, p < 0.001).

**Fig. S5. Differences in the taxonomic diversity of industrialized and non-industrialized human gut microbiomes.** Calculated using Faith's phylogenetic distance for 14 populations of humans (ANOVA F_1,88_ = 95.7, p < 0.001)

**Fig. S6. Differences in the gut microbiome of industrialized and non-industrialized humans. a** Microbial taxa distinguishing human lifestyles. **b** MetaCyc reaction pathways distinguishing human lifestyles.

**Fig. S7. Interindividual variation in the gut microbiome of industrialized and non-industrialized humans.** Principal coordinates analysis (PCoA) plot indicating the distance from each point to the group centroid for **a** taxonomic composition (F_1,88_ = 6.0, p = 0.016) and **b** functional potential (F_1,44_ = 54.8, p < 0.001).

**Fig. S8. Similarity of gut microbiome composition among industrialized and non-industrialized humans, apes, and cercopithecines.** **a** Principal coordinates analysis (PCoA) plot of 16S rRNA gene sequencing data based on unweighted UniFrac distances. **b** PCoA plot of 16S rRNA gene sequencing data based on weighted UniFrac distances. **c** Consensus unweighted pair group method with arithmetic mean (UPGMA) tree of 16S rRNA gene sequencing data based on unweighted UniFrac distances **d** Consensus UPGMA tree of 16S rRNA gene sequencing data based on weighted UniFrac distances**.** PERMANOVA confirms greater differences when comparing humans to apes (unweighted UniFrac: F_1,108_ = 17.0, r^2^ = 0.14, p <0.001; weighted UniFrac: F_1,108_ = 14.8, r^2^ = 0.12, p <0.001) than when comparing humans to cercopithecines (unweighted UniFrac: F_1,117_ = 16.5, r^2^ = 0.12, p <0.001; weighted UniFrac: F_1,117_ = 13.7, r^2^ = 0.11, p <0.001).

**
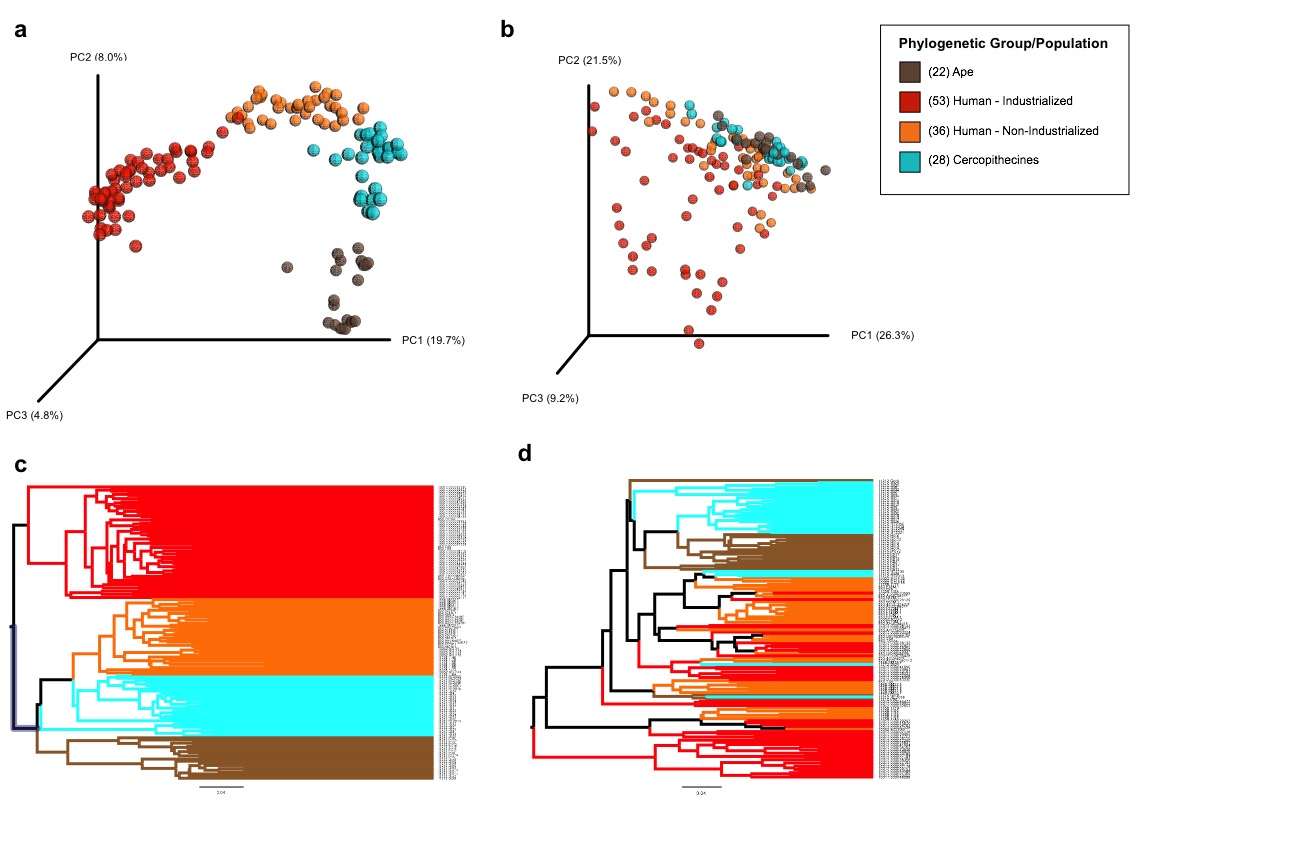
**

**Fig. S9. Similarity of gut microbiome composition among non-industrialized humans, apes, and cercopithecines.** Principal coordinates analysis (PCoA) plot of shotgun metagenomic data describing microbiome taxonomy, based on Bray Curtis distances.

**
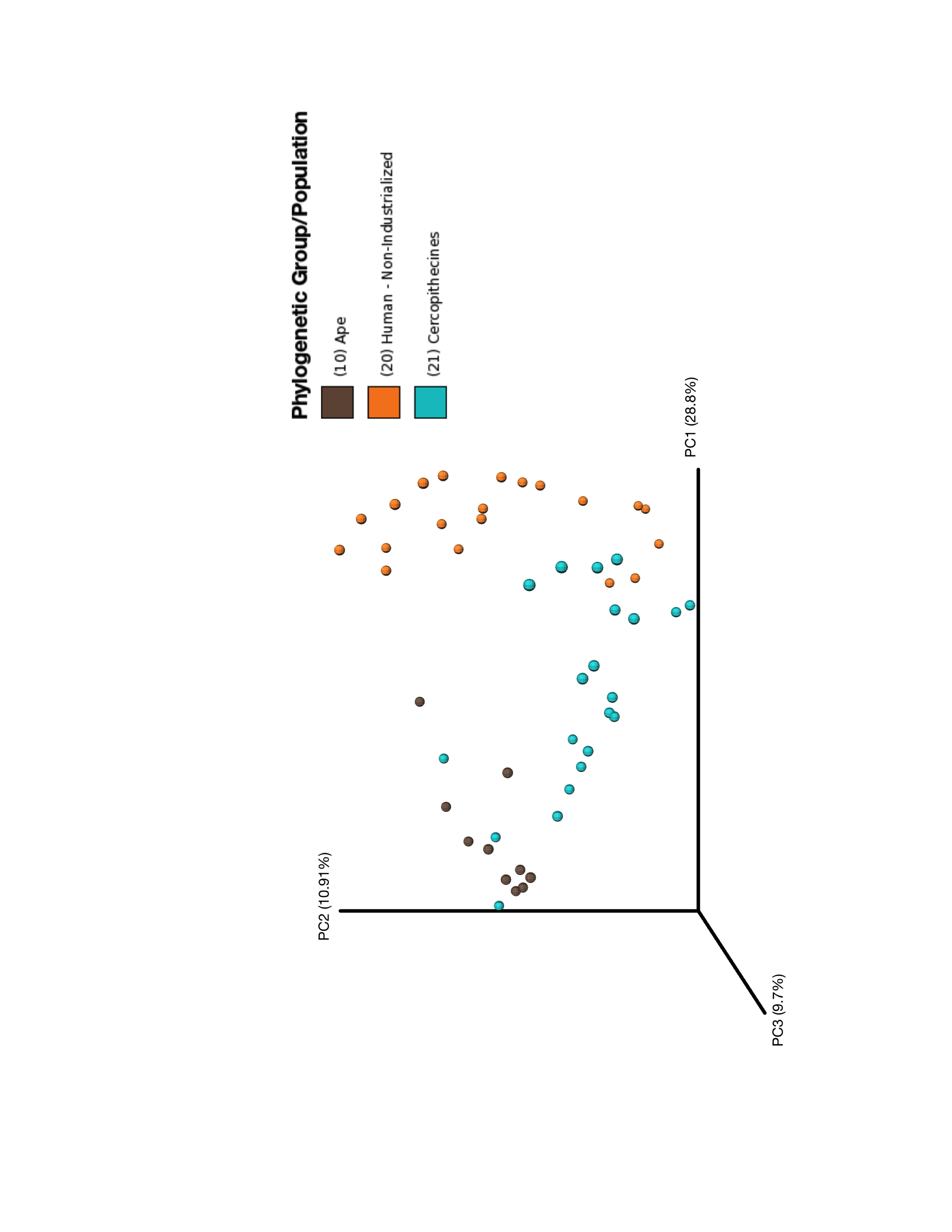
**

**Fig. S10. Microbial taxa distinguishing non-industrialized humans and apes, non-industrialized humans and cercopithecines.** Features with a logarithmic LDA score of >3.0 after LefSe analysis

**Fig. S11.** **Role of baboons in driving similarity of gut microbiome composition among non-industrialized humans, apes, and cercopithecines.** **a** Principal coordinates analysis (PCoA) plot of 16S rRNA gene sequencing data based on unweighted UniFrac distances. Baboons are highlighted by enlarged spheres. **b** PCoA plot of 16S rRNA gene sequencing data based on weighted UniFrac distances. Baboons are highlighted by enlarged spheres. **c** Consensus unweighted pair group method with arithmetic mean (UPGMA) tree of 16S rRNA gene sequencing data based on unweighted UniFrac distances. Baboons are highlighted with colored labels **d** Consensus UPGMA tree of 16S rRNA gene sequencing data based on weighted UniFrac distances. Baboons are highlighted with colored labels.

**Fig. S12. Microbial taxa distinguishing non-industrialized humans and baboons.** Features with a logarithmic LDA score of >3.0 after LefSe analysis.

**
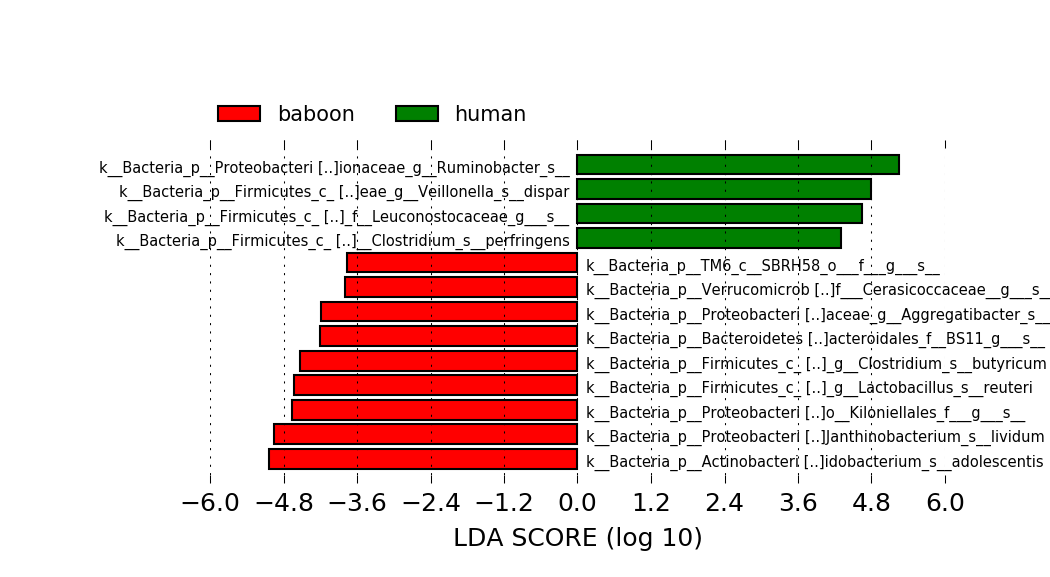
**

**Fig. S13. Similarity of gut microbiome functional potential among all humans, apes, and cercopithecines.** **a** Principal coordinates analysis (PCoA) plot of shotgun metagenomic sequencing data based on Bray-Curtis distances **b** Consensus unweighted pair group method with arithmetic mean (UPGMA) tree of shotgun metagenomic sequencing data based on unweighted UniFrac distances. PERMANOVA indicates greater differences between humans and cercopithecines (Bray-Curtis: F_1,65_ = 13.4, r^2^ = 0.17, p <0.001) than between humans and apes (Bray-Curtis: F_1,54_ = 5.3, r^2^ = 0.09, p = 0.001

**Fig. S14. Metacyc pathways distinguishing non-industrialized humans and apes, non-industrialized humans and cercopithecines.** Features with a logarithmic LDA score of >3.0 after LefSe analysis.

**Fig. S15. Role of baboons in driving similarity of gut microbiome functional potential among non-industrialized humans, apes, and cercopithecines.** **a** Principal coordinates analysis (PCoA) plot of shotgun metagenomic sequencing data based on Bray-Curtis distances. Baboons are highlighted by enlarged spheres. **b** Consensus unweighted pair group method with arithmetic mean (UPGMA) tree of shotgun metagenomic sequencing data based on unweighted UniFrac distances. Baboons are highlighted with colored labels.
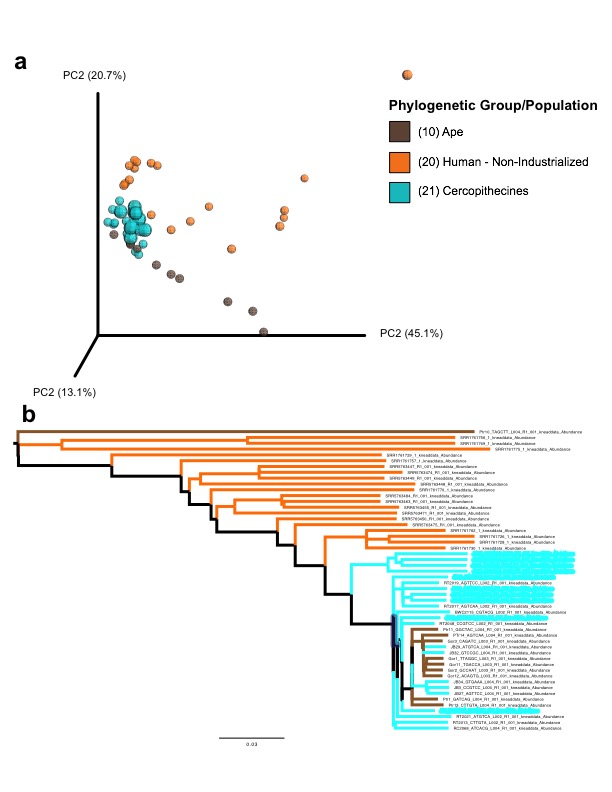


**Fig. S16. Metacyc pathways distinguishing non-industrialized humans and baboons.** Features with a logarithmic LDA score of >3.0 after LefSe analysis.


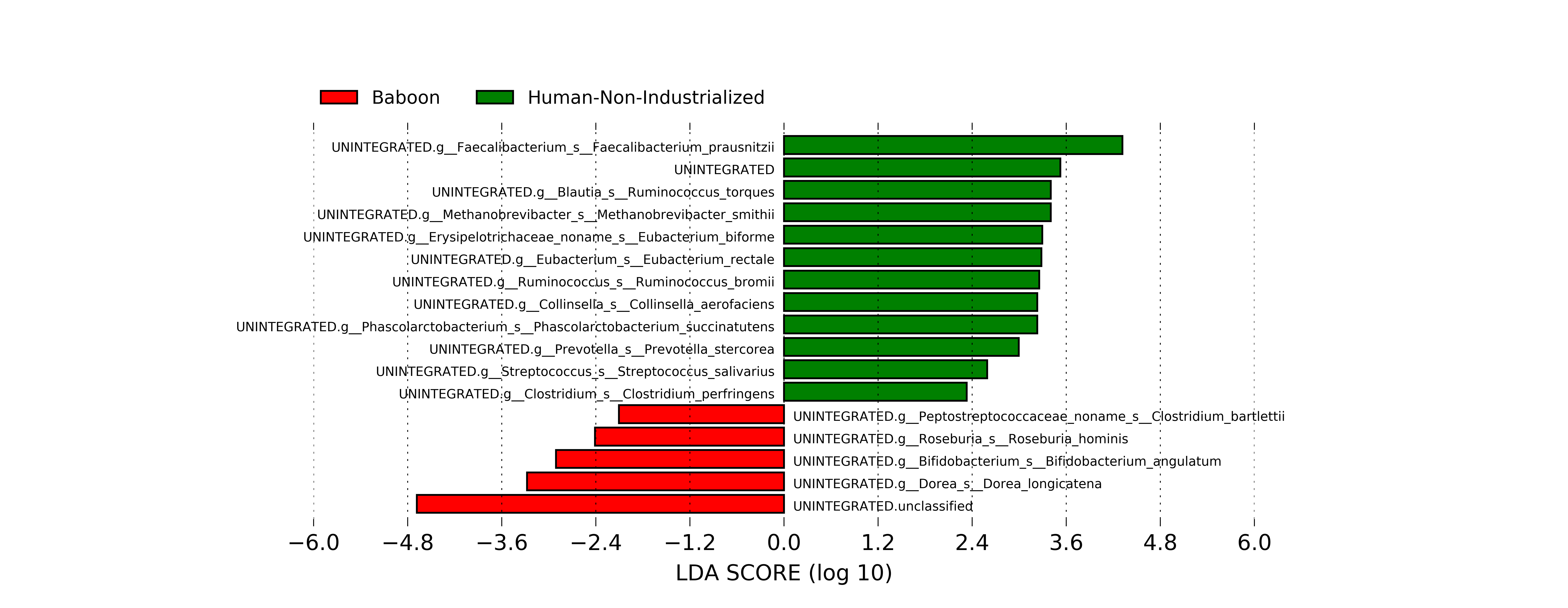


**Fig. S17. Carbohydrate-active enzymes distinguishing non-industrialized humans and apes, non-industrialized humans and cercopithecines.** Features with a logarithmic LDA score of >3.0 after LefSe analysis.

**Fig. S18. Carbohydrate-active enzymes distinguishing non-industrialized humans and baboons.** Features with a logarithmic LDA score of >3.0 after LefSe analysis.**
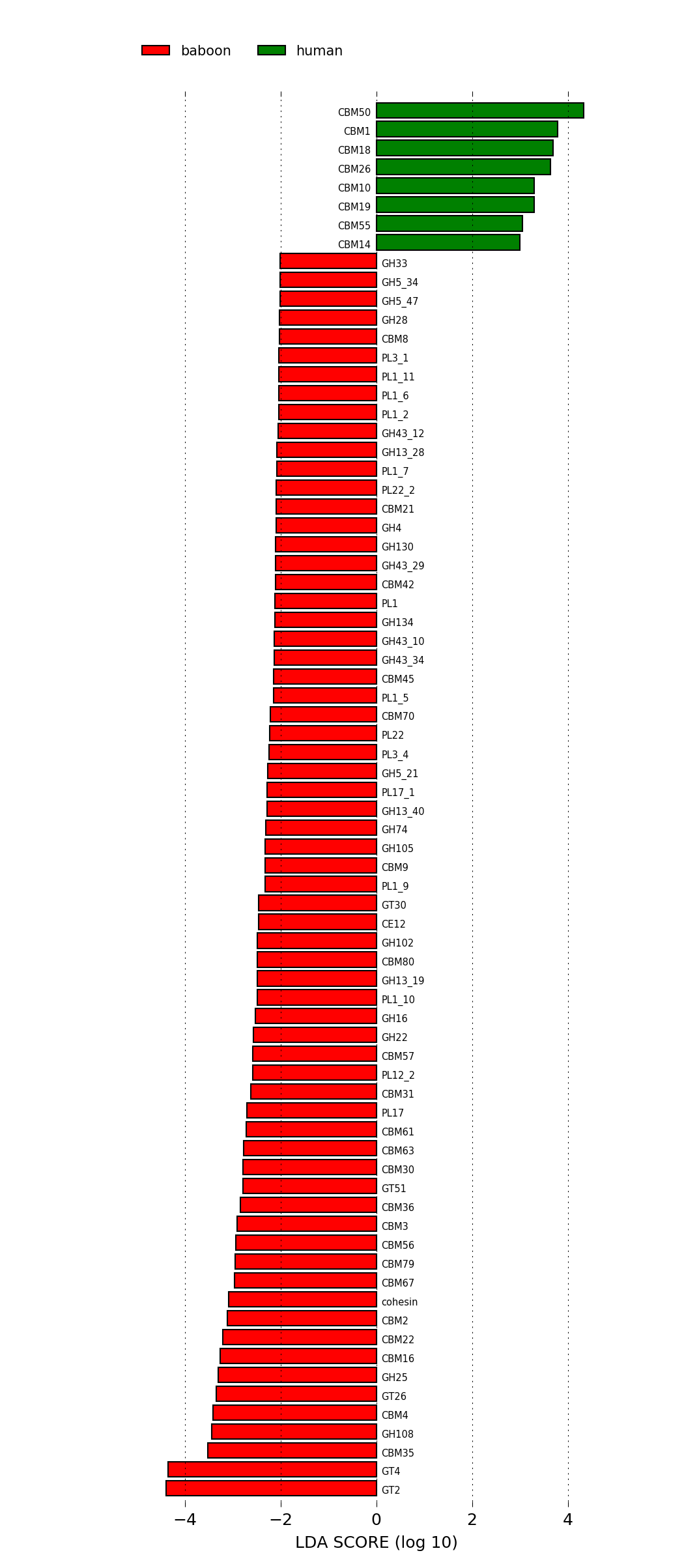
**

**Fig. S19. Microbial taxa distinguishing non-industrialized humans from both apes and cercopithecines.** Features with a logarithmic LDA score of >3.0 after LefSe analysis.

**
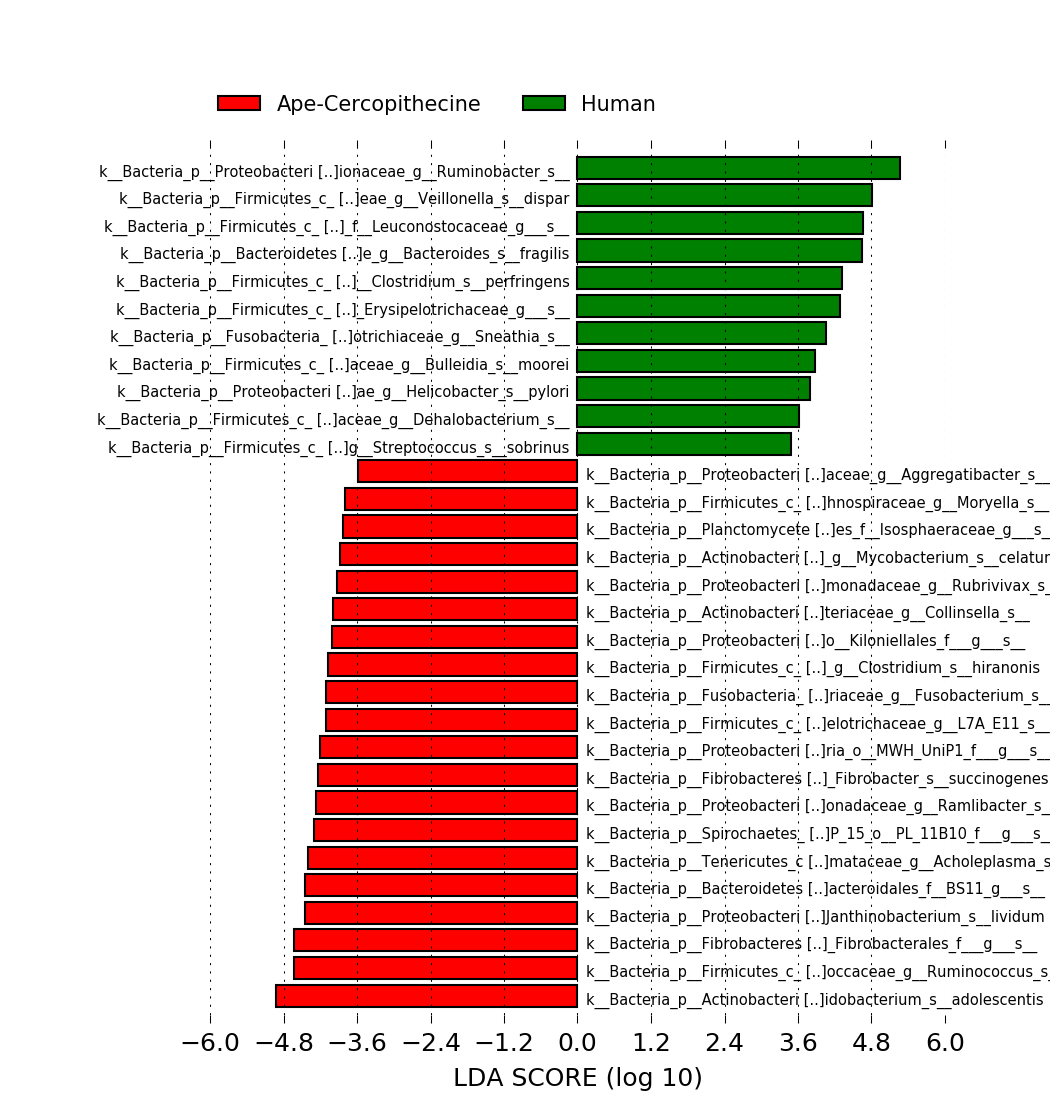
**

**Fig. S20. Metacyc pathways distinguishing non-industrialized humans from both apes and cercopithecines.** Features with a logarithmic LDA score of >3.0 after LefSe analysis.

**
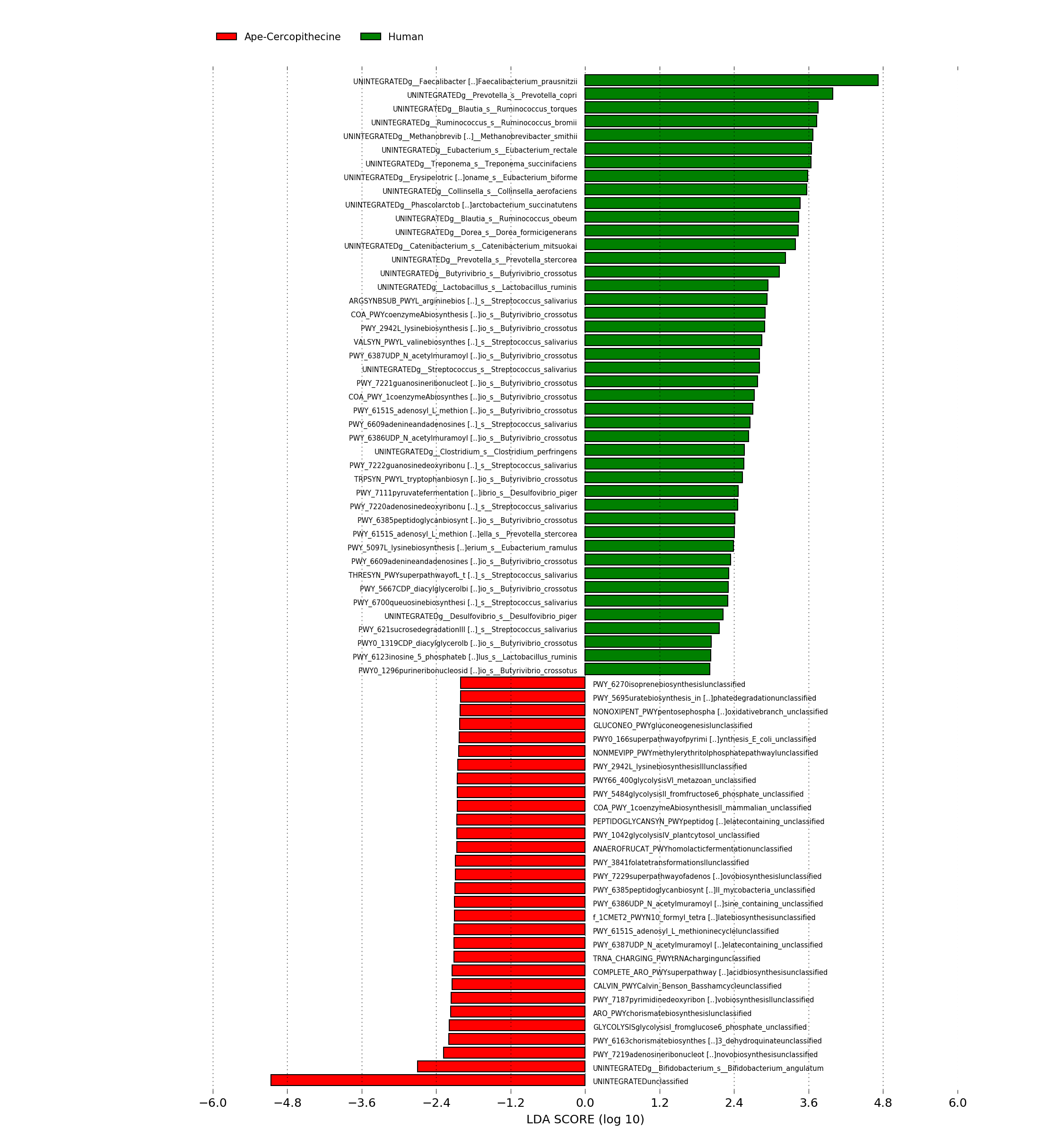
**

**Fig. S21. Carbohydrate-active enzymes distinguishing non-industrialized humans from both apes and cercopithecines.** Features with a logarithmic LDA score of >3.0 after LefSe analysis.

**
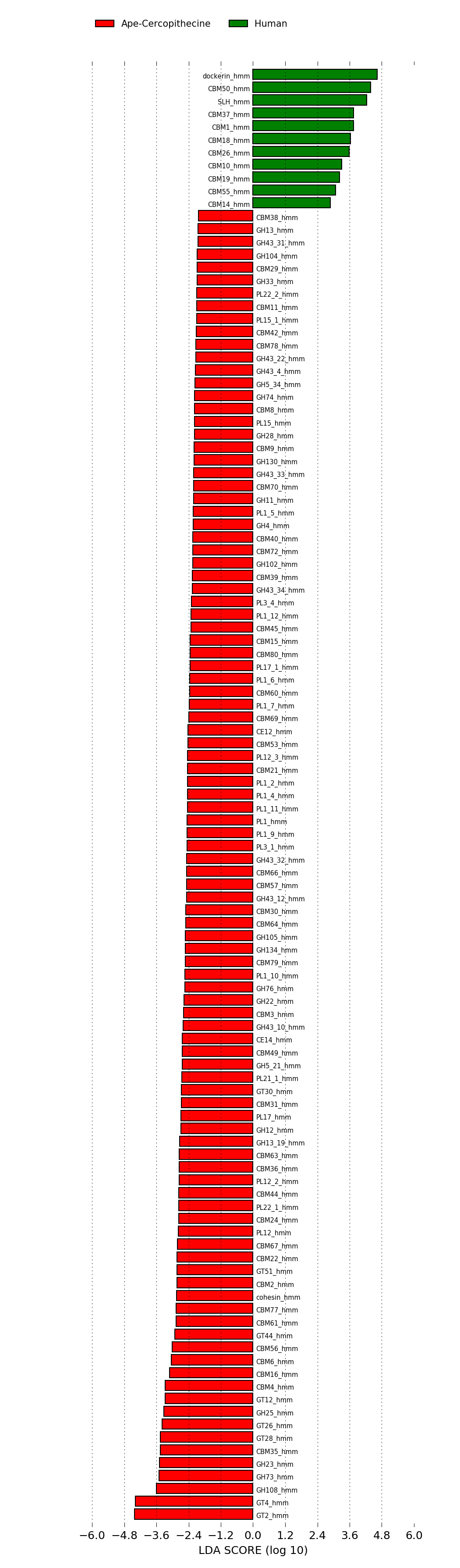
**

**Fig. S22. Interindividual variation in the gut microbiome of non-industrialized humans and apes/cerocpithecines.** Principal coordinates analysis (PCoA) plot indicating the distance from each point to the group centroid for humans and closely related non-human primates for **a** taxonomic composition and **b** functional potential.
