# Supplementary material for: Convergence of human and Old World monkey gut microbiomes demonstrates the importance of human ecology over phylogeny
Source: Genome Biol. 2019 Oct 8;20:201. doi: 10.1186/s13059-019-1807-z (PMC6781418; doi:10.1186/s13059-019-1807-z)
Supplement: Supplementary file 7 — Review history. (DOCX 33 kb) [file 13059_2019_1807_MOESM7_ESM.docx]

Review History

**First round of review**

**Reviewer 1**

**Are you able to assess all statistics in the manuscript, including the appropriateness of statistical tests used?** Yes, and I have assessed the statistics in my report.

**Comments to author:**

The current paper is a follow-up to an earlier paper published recently in ISME J (ref 7 in the current manuscript), in which Amato et al analyzed the gut microbiomes of various primates in the wild (through fecal samples), and provided support for the idea that host phylogeny, and even more so host physiology, structured organisms gut microbiome. In the current paper, the authors use the dataset published in the ISME J paper and use it in a comparison with several human gut microbiome datasets (from industrialized populations as well as from hunter-gatherer or remote farmer communities) to test the hypothesis that host dietary ecology and digestive physiology influence the human gut microbiome independently of host phylogeny. Based on this comparison the authors conclude that it is not host phylogeny per se that structures the microbiota, but rather the accompanying changes in host ecology. They provide support for this conclusion by showing greater similarity between the gut microbiome of humans and cercopithecines/"baboons" (which share more of their ecology and digestive physiology) than between humans and apes (which share more of their phylogeny).

The hypothesis is sound and interesting, and the analysis designed to test it is reasonable. The results seem to support the hypothesis, BUT the organization of the results section makes it hard to see that. The authors begin with a comparison of all human gut microbiomes vs. primates. This does not provide a strong support for their conclusions, since the hypothesis (greater similarity between humans and baboons) is only supported by coordinate analysis using unweighted UNIFRAC distances (and only marginally, with PERMANOVA showing F-value of 16.5 for the humans vs. baboons comparison, and 17.0 for humans and apes); conclusions are not supported by analysis using weighted distances, or by metagenomic functional analysis (Fig. 1b and d, and Fig. 2). In the latter two comparisons all microbiomes in organisms living in a "more ancestral/natural" context are more or less similar, while those of humans of industrialized populations are different. Indeed, when samples from industrialized human populations are removed (as they may represent unnatural conditions, following the same logic that the authors emphasized in their ISME J paper, which should be explained here as well), the trends reported are stronger (Figs. S16, S17), with much larger F value differences, except for the functional analysis, which again points to a greater similarity between apes and humans.

With that in mind, my comments are detailed below:

1) Analysis without industrialized human populations is potentially more accurate and should be highlighted and described earlier in the paper. Furthermore, the expression "…the signal of host phylogeny on gut microbiome becomes more marked" on line 219 is confusing, and should be rephrased. Lastly, Figures S16 and S17 should be put together as one figure in the main text.

2) The authors address the discrepancy (relative to the main conclusions) represented by the results from metagenomic analyses of function, which demonstrate a greater similarity between humans and apes than between humans and baboons, by saying that this this functional similarity is driven by 'housekeeping' genes like cell wall synthesis etc, while pathways associated with digestion are more similar between humans and baboons, in line with the general conclusion. They leave it at that, without considering why would housekeeping genes align more with phylogeny, while niche related genes align more with ecology. They should. One possibility is that housekeeping genes may be associated with core bacteria co-diversifying with hosts, while niche-related digestive genes may be associated with non co-diversifying bacteria. There are of course alternative explanations, but all those should be considered in the discussion.

3) Some explanation is warranted of the significance of F and r squared values from PERMANOVA tests, as these are used frequently in the paper.

4) the section between lines 205-215 is more of a shopping list and lacks a clear take-home message. It should be better summarized.

**Reviewer 2**

**Are you able to assess all statistics in the manuscript, including the appropriateness of statistical tests used?** Yes, and I have assessed the statistics.

**Comments to author:**

This is an interesting manuscript describing the comparative analysis of the fecal microbiome of 14 distinct human populations and that of 18 wild primates with the goal to gain insight into the processes that shaped the evolution of the human gut microbiome. The overall rationale of the work is well justified and the conceptual ideas are innovative, and several of the findings are clearly of interest. I am a bit underwhelmed by the presentation of the findings and their interpretations, and the overall writing (e.g. the discussion), and I wonder how confounded the analysis is through the use of datasets obtained in different studies with different designs.

Major points:

1.     Presentation and Interpretations: Some of the key conclusions of the findings, for example that the human microbiomes are more similar to cercopithecines, are based more on descriptive observations and not on rigid statistical tests. Differences between groups were only analysed by PERMANOVA, and conclusions on the differences between these intra-group distances (human to ape versus human to cercopithecines) were then based on comparisons of F values and effect sizes (meaning, effect sizes are larger or smaller in one intra-group comparison to another). First, these measures (F, r2) are never explained but really should be as much of the interpretation is based on them, and second, it seems that differences between intra-group distances, which matter most for the objective of the study (for example, differences between humans and apes versus humans and cercopithecines), were never rigidly tested. The same also accounts for the number of genes that are shared among groups (see specific points below). In this respect, it often seems a bit arbitrary which analyses are presented (or were done) and which not. For example, taxonomic diversity analysis was only done with the 16S amplicon data but not the metagenomics data, although it would be interesting to see if the findings match (e.g. since metagenomic data is less susceptible to confounders through study and batch effects). The manuscript reads as if the authors did a broader analysis of the overall data, came to a conclusion, and then selectively chose hypotheses and analyses to support these conclusions. Although I understand that such an approach is often done when analysing larger sequence dataset, and I am in general agreement with the interpretations of the findings, a more systematic and robust approach in actually testing hypotheses would improve the manuscript.

2.     Writing: The language throughout the manuscript is a bit vague (see specific points below), and the manuscript could be improved in how it discusses the findings in light of the available literature. For example, not even the own work of the first author, which does contradict some of the findings, is sufficiently discussed, and there are other examples (e.g. distinct diversity patterns between industrialized and non-industrialized human populations, and even what might cause these patterns, are well covered in the literature but this is not adequately addressed). Also, some interesting findings should be discussed in the light of ecological and evolutionary concepts. What stood out to me was the findings that humans and apes are more similar when it comes to house keeping functions, while humans and cercopithecines are more similar when it comes to arguably more relevant functions for host biology. How could this evolve, or did it even evolve or is just a reflection of recent changes in human ecology? All of this is in the manuscript but I thought it could be better explained.

3.     Confounders: I know it is common practice to compare data from different studies. However, the authors should at least provide a disclaimer on why they do not think that their conclusions are not impacted by their multi-study design. I have seen samples cluster by sequence run even within one study. Other confounders that should be discussed in greater detail is the use of diverse human populations, but tighter groups of monkeys. How would differences in inter-group variation even among the monkeys confound the findings?

Specific points:

Line 42: What would a 'typical' microbiome be? I understand that it is difficult to define, but I do not even think there is a hypothetical concept for it. So I do not think it is helpful to throw this into the abstract without explaining it.

Line 44: What are 'microbial traits' in this context? As used here, this is vague. Evolution of the microbiome might result in the host selecting for communities with specific features, composed of microbes with specific phenotypic traits. As written, I can guess but I do not really know what the authors mean.

Line 52: More vague language. What is 'flexibility' of a microbiome?

Line 72: True that it is difficult to define a healthy microbiome, but this work will not contribute to this, and as I state above, I do not know what a 'typical' microbiome would be.

Lines 76-77: In what sense is a mammalian microbiome 'unique'?

Line 78: What traits? And how does that help to define a healthy microbiome? We are trying this for 15 years now, and whole NIH programs were devoted on it, and we were not successful (and might not be successful). The authors are advised to focus their introduction on specific findings from their study, which are many, and not on vague concepts on microbiome health.

Line 80: 'Microbial traits' is vague. These could be 'microbiome traits' (diversity, composition), or traits of the microbes, or both.

Lines 86-87: Avoid slang. It is not the microbes that co-diversify, but microbial species, taxa, lineages, or populations.

Line 89: Can you add what you mean by 'majority'? >80%? >90%?

Lines 89-96: I agree what is written there (or at least I think I do), but find the language and terminology vague throughout this paragraph. Just one example: What are 'host ecological traits'?

Line 101: I would not use the term 'quality'? If necessary, then please define in what respect the diet is of high quality. Meet is probably more 'nutritious'.

Lines 108-109: How can these apes consume fruit 'regardless of availability'. If fruit is not available, they cannot consume it. Do the authors mean that these apes consume a high percentage of fruits even if they have alternative options?

Line 113: I struggle with the term 'ecological trait'. Please define or change to physiological traits that reflect human ecology'.

Lines 115-116: Please rewrite. Primates cannot be an analogue for hominin evolution. The way they evolved might be analogous to how humans evolved.

Lines 146-151: I find it extremely weird to begin the result section with what is essentially the central conclusion of the work.

Lines 158-165: The greater differences of the microbiome from human to apes versus humans to cercopithecines is one of the central findings of the study (and super interesting). However, it is not well presented. As I understand it here, this conclusion is based on comparing F and r2 values from two statistical analyses (which were different but both highly significant), but not through a statistical test that actually tests the hypothesis. I would like to see the average distances between humans and apes and humans and cercopithecines compared, similar to what the authors did for variation of microbiomes in Figure S21. I would like to see this both for Unifrac and Bray Curtis, as the two distances show different things.

Lines 164-168: Same thing here. It is interesting that humans and cercopithecies share more OTUs, but it is not clear that this difference reaches significance. Please test.

Lines 175-178: Same here. Why not test if average differences are statistically significant?

Line 181-187: Please provide the data here with statistical tests. Apart from the lack of statistical tests that actually test hypothesis, the presentation of the data is rather vague here.

Line 193-196: Again, please test if these differences are significant.

Line 199-200: Before reading this manuscript, I would find this surprising.

Line 207: Define sOTUs.

Lines 217-218: Provide references.

Line 243: Define what 'the rest' is. Was 'the rest' even studied? There are a lot of primate species. Also, provide reference for this statement (I think that is the first author's own work).

Line 246: References?

Line 249: Again, provide references. There are strong statements here that should be referenced.

Line 249-250: 'existing' is not a good word here. Even if the lineages would be stably maintained throughout evolution, they would still have 'existed'.

Line 255: Rewrite. Would that not just be 'biogeography'? Use ecological terms!

Line 270: Rewrite 'ape-like microbial qualities'? This is slang. The microbial qualities are really not ape like.

Lines 285-292: This is starting to be rather well studied, but the authors make it sound as if they discovered this, and not one single reference is provided. Also, ecological concepts have been suggested to provide an explanation (see here for example: https://elifesciences.org/articles/36521).

**Authors Response**

We thank both reviewers for their helpful comments on this manuscript. We have addressed all concerns and find the paper to substantially improved. We hope that the writing is now easier to follow and presents a clearer message. We comment on specific suggestions below in blue.

Reviewer #1: The current paper is a follow-up to an earlier paper published recently in ISME J (ref 7 in the current manuscript), in which Amato et al analyzed the gut microbiomes of various primates in the wild (through fecal samples), and provided support for the idea that host phylogeny, and even more so host physiology, structured organisms gut microbiome. In the current paper, the authors use the dataset published in the ISME J paper and use it in a comparison with several human gut microbiome datasets (from industrialized populations as well as from hunter-gatherer or remote farmer communities) to test the hypothesis that host dietary ecology and digestive physiology influence the human gut microbiome independently of host phylogeny. Based on this comparison the authors conclude that it is not host phylogeny per se that structures the microbiota, but rather the accompanying changes in host ecology. They provide support for this conclusion by showing greater similarity between the gut microbiome of humans and cercopithecines/"baboons" (which share more of their ecology and digestive physiology) than between humans and apes (which share more of their phylogeny). 

The hypothesis is sound and interesting, and the analysis designed to test it is reasonable. The results seem to support the hypothesis, BUT the organization of the results section makes it hard to see that. The authors begin with a comparison of all human gut microbiomes vs. primates. This does not provide a strong support for their conclusions, since the hypothesis (greater similarity between humans and baboons) is only supported by coordinate analysis using unweighted UNIFRAC distances (and only marginally, with PERMANOVA showing F-value of 16.5 for the humans vs. baboons comparison, and 17.0 for humans and apes); conclusions are not supported by analysis using weighted distances, or by metagenomic functional analysis (Fig. 1b and d, and Fig. 2). In the latter two comparisons all microbiomes in organisms living in a "more ancestral/natural" context are more or less similar, while those of humans of industrialized populations are different. Indeed, when samples from industrialized human populations are removed (as they may represent unnatural conditions, following the same logic that the authors emphasized in their ISME J paper, which should be explained here as well), the trends reported are stronger (Figs. S16, S17), with much larger F value differences, except for the functional analysis, which again points to a greater similarity between apes and humans. 

With that in mind, my comments are detailed below: 
1) Analysis without industrialized human populations is potentially more accurate and should be highlighted and described earlier in the paper. Furthermore, the expression "…the signal of host phylogeny on gut microbiome becomes more marked" on line 219 is confusing, and should be rephrased. Lastly, Figures S16 and S17 should be put together as one figure in the main text.

RESPONSE: Thank you for this suggestion and for the more general evaluation of the paper above. We agree with the review and have now re-organized the manuscript to highlight the differences between human populations first, providing rationale for removing the industrialized humans from the analysis. All subsequent analyses focus on non-industrialized humans, which makes the paper much cleaner and easier to read. Select results for analyses including industrialized humans are still included in the supplemental material. We also note that we have altered the supplemental figures and changed the analysis and figure that was previously associated with Figures S16 and S17 (now S22). We are happy to move this figure to the main text if still deemed appropriate.

2) The authors address the discrepancy (relative to the main conclusions) represented by the results from metagenomic analyses of function, which demonstrate a greater similarity between humans and apes than between humans and baboons, by saying that this this functional similarity is driven by 'housekeeping' genes like cell wall synthesis etc, while pathways associated with digestion are more similar between humans and baboons, in line with the general conclusion. They leave it at that, without considering why would housekeeping genes align more with phylogeny, while niche related genes align more with ecology. They should. One possibility is that housekeeping genes may be associated with core bacteria co-diversifying with hosts, while niche-related digestive genes may be associated with non co-diversifying bacteria. There are of course alternative explanations, but all those should be considered in the discussion.

RESPONSE: These results have shifted slightly given the removal of industrialized humans, and differences in the shotgun data between humans and cercopithecines are no longer as strong. However, we still feel there is a similar pattern in the data and we discuss the potential mechanisms behind it in the revised discussion.

3) Some explanation is warranted of the significance of F and r squared values from PERMANOVA tests, as these are used frequently in the paper.

RESPONSE: We did not originally deem this necessary given that the statistics are similar to those used with other tests. We now include a sentence that addresses this in the results, the first time the statistics are reported. We also address this in the methods.

4) the section between lines 205-215 is more of a shopping list and lacks a clear take-home message. It should be better summarized.

RESPONSE: Thank you for pointing this out. This section has been revised.


Reviewer #2: This is an interesting manuscript describing the comparative analysis of the fecal microbiome of 14 distinct human populations and that of 18 wild primates with the goal to gain insight into the processes that shaped the evolution of the human gut microbiome. The overall rationale of the work is well justified and the conceptual ideas are innovative, and several of the findings are clearly of interest. I am a bit underwhelmed by the presentation of the findings and their interpretations, and the overall writing (e.g. the discussion), and I wonder how confounded the analysis is through the use of datasets obtained in different studies with different designs. 

Major points: 
1.	Presentation and Interpretations: Some of the key conclusions of the findings, for example that the human microbiomes are more similar to cercopithecines, are based more on descriptive observations and not on rigid statistical tests. Differences between groups were only analysed by PERMANOVA, and conclusions on the differences between these intra-group distances (human to ape versus human to cercopithecines) were then based on comparisons of F values and effect sizes (meaning, effect sizes are larger or smaller in one intra-group comparison to another). First, these measures (F, r2) are never explained but really should be as much of the interpretation is based on them, and second, it seems that differences between intra-group distances, which matter most for the objective of the study (for example, differences between humans and apes versus humans and cercopithecines), were never rigidly tested. The same also accounts for the number of genes that are shared among groups (see specific points below). In this respect, it often seems a bit arbitrary which analyses are presented (or were done) and which not. For example, taxonomic diversity analysis was only done with the 16S amplicon data but not the metagenomics data, although it would be interesting to see if the findings match (e.g. since metagenomic data is less susceptible to confounders through study and batch effects). The manuscript reads as if the authors did a broader analysis of the overall data, came to a conclusion, and then selectively chose hypotheses and analyses to support these conclusions. Although I understand that such an approach is often done when analysing larger sequence dataset, and I am in general agreement with the interpretations of the findings, a more systematic and robust approach in actually testing hypotheses would improve the manuscript. 

RESPONSE: We thank the reviewer for the assessments above. We agree with many of these points and have analyzed the manuscript accordingly. First, as explained above, we now include a sentence explaining what F and r2 values are. They are quite similar to other F and r2 values most readers will have seen in the past. Second, we have added more statistical analyses, as suggested (e.g., t-test of inter-group differences, assessment of taxonomic composition using shotgun data). We appreciate that the manuscript was not clearly written, but we would like to point out that we did present all of the analyses that we did and were not selectively presenting data. In fact, that is likely what contributed to some of the confusion. As the other reviewer suggested, we have now narrowed the analyses to non-industrialized humans only which greatly clarifies the message. We also thought the broader analyses provided important context within the entire primate phylogeny and so have kept this in the paper.

2.	Writing: The language throughout the manuscript is a bit vague (see specific points below), and the manuscript could be improved in how it discusses the findings in light of the available literature. For example, not even the own work of the first author, which does contradict some of the findings, is sufficiently discussed, and there are other examples (e.g. distinct diversity patterns between industrialized and non-industrialized human populations, and even what might cause these patterns, are well covered in the literature but this is not adequately addressed). Also, some interesting findings should be discussed in the light of ecological and evolutionary concepts. What stood out to me was the findings that humans and apes are more similar when it comes to house keeping functions, while humans and cercopithecines are more similar when it comes to arguably more relevant functions for host biology. How could this evolve, or did it even evolve or is just a reflection of recent changes in human ecology? All of this is in the manuscript but I thought it could be better explained.

RESPONSE: Thank you for pointing this gap out. We agree that the study was not properly referenced and have no explanation. We have fixed this. We also discuss the patterns in the shotgun data in somewhat more detail in the revised discussion.

3.	Confounders: I know it is common practice to compare data from different studies. However, the authors should at least provide a disclaimer on why they do not think that their conclusions are not impacted by their multi-study design. I have seen samples cluster by sequence run even within one study. Other confounders that should be discussed in greater detail is the use of diverse human populations, but tighter groups of monkeys. How would differences in inter-group variation even among the monkeys confound the findings?

RESPONSE: The reviewer is correct that this is often an issue. We purposely only chose studies that used the same or very similar lab protocols (especially primers), and in fact, many of the data presented were generated by the same lab. Therefore, we think that inter-study confounds are relatively small. We also detected no effect of technical variables. We now discuss this more in the discussion.

Specific points: 

Line 42: What would a 'typical' microbiome be? I understand that it is difficult to define, but I do not even think there is a hypothetical concept for it. So I do not think it is helpful to throw this into the abstract without explaining it. 
Line 44: What are 'microbial traits' in this context? As used here, this is vague. Evolution of the microbiome might result in the host selecting for communities with specific features, composed of microbes with specific phenotypic traits. As written, I can guess but I do not really know what the authors mean. 
Line 52: More vague language. What is 'flexibility' of a microbiome? 
Line 72: True that it is difficult to define a healthy microbiome, but this work will not contribute to this, and as I state above, I do not know what a 'typical' microbiome would be. 
Lines 76-77: In what sense is a mammalian microbiome 'unique'? 
Line 78: What traits? And how does that help to define a healthy microbiome? We are trying this for 15 years now, and whole NIH programs were devoted on it, and we were not successful (and might not be successful). The authors are advised to focus their introduction on specific findings from their study, which are many, and not on vague concepts on microbiome health. 
Line 80: 'Microbial traits' is vague. These could be 'microbiome traits' (diversity, composition), or traits of the microbes, or both. 
Lines 86-87: Avoid slang. It is not the microbes that co-diversify, but microbial species, taxa, lineages, or populations.
Line 89: Can you add what you mean by 'majority'? >80%? >90%? 
Lines 89-96: I agree what is written there (or at least I think I do), but find the language and terminology vague throughout this paragraph. Just one example: What are 'host ecological traits'? 
Line 101: I would not use the term 'quality'? If necessary, then please define in what respect the diet is of high quality. Meet is probably more 'nutritious'. 
Lines 108-109: How can these apes consume fruit 'regardless of availability'. If fruit is not available, they cannot consume it. Do the authors mean that these apes consume a high percentage of fruits even if they have alternative options? 
Line 113: I struggle with the term 'ecological trait'. Please define or change to physiological traits that reflect human ecology'. 
Lines 115-116: Please rewrite. Primates cannot be an analogue for hominin evolution. The way they evolved might be analogous to how humans evolved.

RESPONSE: We have addressed all of these points in the revision and hope the language is now clearer.

Lines 146-151: I find it extremely weird to begin the result section with what is essentially the central conclusion of the work.

RESPONSE: We agree although some journals like this. It has been removed.

Lines 158-165: The greater differences of the microbiome from human to apes versus humans to cercopithecines is one of the central findings of the study (and super interesting). However, it is not well presented. As I understand it here, this conclusion is based on comparing F and r2 values from two statistical analyses (which were different but both highly significant), but not through a statistical test that actually tests the hypothesis. I would like to see the average distances between humans and apes and humans and cercopithecines compared, similar to what the authors did for variation of microbiomes in Figure S21. I would like to see this both for Unifrac and Bray Curtis, as the two distances show different things.

RESPONSE: PERMANOVA should address this comparison. We also ran an envfit model in the first version of the manuscript, which tests the fit of the 'phylogeny' and 'ecology' vectors simultaneously in the same model. These results remain in this version of the manuscript. We have now also included a t-test comparing the inter-group distances of humans and apes and humans and cercopithecines. Bray-Curtis distances give similar results as do the shotgun metagenomic data. To avoid spending space on iterations of similar tests, we present the Bray Curtis results for the shotgun data only, included in the supplemental data.

Lines 164-168: Same thing here. It is interesting that humans and cercopithecies share more OTUs, but it is not clear that this difference reaches significance. Please test.

RESPONSE: This finding was determined by calculating the 'core' microbiome, taxa that are prevalent in 80% of the group of samples included in the analysis. Given that this is a presence/absence evaluation, we are not sure how one would statistically test this further aside from saying, as we did, that a certain group of taxa were prevalent in 80% of the samples for whatever two groups we were testing.

Lines 175-178: Same here. Why not test if average differences are statistically significant?

RESPONSE: We now do this as explained above.

Line 181-187: Please provide the data here with statistical tests. Apart from the lack of statistical tests that actually test hypothesis, the presentation of the data is rather vague here.

RESPONSE: We have revised this section and hope it is clearer.

Line 193-196: Again, please test if these differences are significant.

RESPONSE: We continue to include the statistical tests that were already present and added the t test of inter-group distances.

Line 199-200: Before reading this manuscript, I would find this surprising. 
Line 207: Define sOTUs. 
Lines 217-218: Provide references. 
Line 243: Define what 'the rest' is. Was 'the rest' even studied? There are a lot of primate species. Also, provide reference for this statement (I think that is the first author's own work). 
Line 246: References? 
Line 249: Again, provide references. There are strong statements here that should be referenced. 
Line 249-250: 'existing' is not a good word here. Even if the lineages would be stably maintained throughout evolution, they would still have 'existed'. 
Line 255: Rewrite. Would that not just be 'biogeography'? Use ecological terms!
Line 270: Rewrite 'ape-like microbial qualities'? This is slang. The microbial qualities are really not ape like. 
Lines 285-292: This is starting to be rather well studied, but the authors make it sound as if they discovered this, and not one single reference is provided. Also, ecological concepts have been suggested to provide an explanation (see here for example: <https://urldefense.proofpoint.com/v2/url?u=https-3A__elifesciences.org_articles_36521&d=DwIGaQ&c=yHlS04HhBraes5BQ9ueu5zKhE7rtNXt_d012z2PA6ws&r=LM09evdnoYZOSKkcw8zx0UFHZyITSrLcY0F_toUlR2I&m=VPhvi3ygbUAs2H16N5LZCjPSgtTvJc5GOgRlYJmnZgg&s=wu1zMfgo5ggFdU1rWYLITKdHqnxoV-E6UJU7g_UCXqM&e=>).

RESPONSE: We have addressed all of the above issues.

**Second round of review**

**Reviewer 1**

The paper has somewhat improved, but it is still not focused enough, and the writing is not clear enough. The way it is written equally highlights what appears to be trivial points and important findings. Explanation of methodology is also lacking.

The authors tried to shorten the part of the analysis that included industrialized human populations, but still take the reader through a detailed description of these analyses, before getting to the main results. This includes detailed comparisons of industrialized and non-industrialized human populations (seven supplementary figures, plus all the statistics in the main text), which are not the subject of the current paper, and were published and discussed by others before.

Why can't they just cut to the chase, by saying something like "We first compared gut microbiome composition and function in both industrialized and nonindustrialized humans to that of all 18 species of wild primates." … In agreement with previous reports, industrialized and non-industrialized human populations differed significantly (both based on 16S data as well as metagenomic analysis) with non-industrialized populations clustering together with apes and old world monkeys, while gut microbiomes from industrialized populations clustered away from all other microbiomes. To focus analysis on populations living under natural conditions, we removed samples from industrialized human populations from all further analyses. Repeating the analysis with only non-industrialized human populations showed … (Fig. 1).

Line 146: I am not clear why was it necessary to ensure that gut microbiomes in human populations were different from those of new world monkeys and lemurs. It seems quite trivial to me, and not of high priority as suggested by the current position.

Line 177: Explanations of F value and r(sq) should be moved to methods.

Line 178: what does it mean "When we fit and compared vectors of environmental data to the ordination"? Please rephrase in simple language.

Line 190: the authors need to briefly clarify the 85% cutoff used for considering only the core microbiome. In methods, this deserves a fuller explanation.

Line 200: "a similar pattern emerged for a subset of the data." Too ambiguous. This should be rephrased.

The authors report that "The overall dataset indicated slightly greater differences between humans and cercopithecines (line 203), but "Bray Curtis distances between humans and cercopithecines were significantly smaller" (line 208). Could they explain what's the difference between these two comparisons, and what is the significance?

Line 211: again, better explanation of the use of core microbiome

A concluding sentence is in place after line 234, explaining the significance of these findings.

A long and cumbersome sentence begins on line 244 and goes all the way till the end of the paragraph: "Additionally, tests of beta dispersion indicated that while inter-individual variation the taxonomic composition of human and ape/cercopithecine gut microbiomes were similar (Fig. S22, ANOVA F1,83 = 1.4, p = 0.2), inter-individual variation in the functional potential of the human gut microbiome was much greater than that of apes-cercopithecines (Figs. S22; ANOVA F1,49 = 15.2, p < 0.001)." Yet, what it means is not clear. Please rephrase, and add a concluding sentence.

**Reviewer 2**

I am happy with this revision

**Authors Response**

**Point-by-point responses to the reviewers’ comments:**

Reviewer #1: The paper has somewhat improved, but it is still not focused enough, and the writing is not clear enough. The way it is written equally highlights what appears to be trivial points and important findings. Explanation of methodology is also lacking.

The authors tried to shorten the part of the analysis that included industrialized human populations, but still take the reader through a detailed description of these analyses, before getting to the main results. This includes detailed comparisons of industrialized and non-industrialized human populations (seven supplementary figures, plus all the statistics in the main text), which are not the subject of the current paper, and were published and discussed by others before. Why can't they just cut to the chase, by saying something like "We first compared gut microbiome composition and function in both industrialized and nonindustrialized humans to that of all 18 species of wild primates." … In agreement with previous reports, industrialized and non-industrialized human populations differed significantly (both based on 16S data as well as metagenomic analysis) with non-industrialized populations clustering together with apes and old world monkeys, while gut microbiomes from industrialized populations clustered away from all other microbiomes. To focus analysis on populations living under natural conditions, we removed samples from industrialized human populations from all further analyses. Repeating the analysis with only non-industrialized human populations showed … (Fig. 1).

**Response:** We have included the supplemental figures still but revised the text to mirror what the reviewer has suggested almost completely. We agree that this streamlines the paper much better.

Line 146: I am not clear why was it necessary to ensure that gut microbiomes in human populations were different from those of new world monkeys and lemurs. It seems quite trivial to me, and not of high priority as suggested by the current position.

**Response:** This statement has been eliminated with the revisions suggested above.

Line 177: Explanations of F value and r(sq) should be moved to methods.

**Response:** Done.

Line 178: what does it mean "When we fit and compared vectors of environmental data to the ordination"? Please rephrase in simple language.

**Response:** Done

Line 190: the authors need to briefly clarify the 85% cutoff used for considering only the core microbiome. In methods, this deserves a fuller explanation.

**Response:** We have addressed this in the methods as well as briefly here. This should have been listed as 80%, and we chose to use this more relaxed definition of a core given our use of sub-OTU level data across distinct host species that are likely to have more distinct gut microbiomes than individuals of the same host.

Line 200: "a similar pattern emerged for a subset of the data." Too ambiguous. This should be rephrased.

**Response:** We have revised this sentence to more clearly state the overall take-away of this section.

The authors report that "The overall dataset indicated slightly greater differences between humans and cercopithecines (line 203), but "Bray Curtis distances between humans and cercopithecines were significantly smaller" (line 208). Could they explain what's the difference between these two comparisons, and what is the significance?

**Response:** The PERMANOVA is only comparing samples from pairs of host species and is showing the magnitude of difference between within-group and between-group distances in each case. The t test is directly comparing between-group distances for human-cercopithecine and human-ape. We have altered the wording to try to make this clearer.

Line 211: again, better explanation of the use of core microbiome

**Response:** This cutoff should have been the same as the one for 16S so we have acknowledged that in the text and limited the explanation to the first instance.

A concluding sentence is in place after line 234, explaining the significance of these findings.

**Response:** We have included some more text explaining why these findings matter.

A long and cumbersome sentence begins on line 244 and goes all the way till the end of the paragraph: "Additionally, tests of beta dispersion indicated that while inter-individual variation the taxonomic composition of human and ape/cercopithecine gut microbiomes were similar (Fig. S22, ANOVA F1,83 = 1.4, p = 0.2), inter-individual variation in the functional potential of the human gut microbiome was much greater than that of apes-cercopithecines (Figs. S22; ANOVA F1,49 = 15.2, p < 0.001)." Yet, what it means is not clear. Please rephrase, and add a concluding sentence.

**Response:** We have revised this sentence to clarify and added a concluding sentence.
